# Improved Water–Gas Shift Performance of Au/NiAl LDHs Nanostructured Catalysts via CeO_2_ Addition

**DOI:** 10.3390/nano11020366

**Published:** 2021-02-02

**Authors:** Margarita Gabrovska, Ivan Ivanov, Dimitrinka Nikolova, Jugoslav Krstić, Anna Maria Venezia, Dorel Crişan, Maria Crişan, Krassimir Tenchev, Vasko Idakiev, Tatyana Tabakova

**Affiliations:** 1 Institute of Catalysis, Bulgarian Academy of Sciences, 1113 Sofia, Bulgaria; bogoev@ic.bas.bg (I.I.); dimi_nik@abv.bg (D.N.); tenchev@ic.bas.bg (K.T.); idakiev@ic.bas.bg (V.I.); 2 Department of Catalysis and Chemical Engineering, Institute of Chemistry, Technology and Metallurgy, University of Belgrade, 11000 Belgrade, Serbia; jkrstic@nanosys.ihtm.bg.ac.rs; 3 Istituto per lo Studio dei Materiali Nanostrutturati, CNR, 90146 Palermo, Italy; annamaria.venezia@cnr.it; 4 Ilie Murgulescu Institute of Physical Chemistry, Romanian Academy, 060021 Bucharest, Romania; dcrisan@icf.ro (D.C.); mcrisan@icf.ro (M.C.)

**Keywords:** Ni-Al layered double hydroxides, gold catalyst, CeO_2_ addition, water–gas shift reaction

## Abstract

Supported gold on co-precipitated nanosized NiAl layered double hydroxides (LDHs) was studied as an effective catalyst for medium-temperature water–gas shift (WGS) reaction, an industrial catalytic process traditionally applied for the reduction in the amount of CO in the synthesis gas and production of pure hydrogen. The motivation of the present study was to improve the performance of the Au/NiAl catalyst via modification by CeO_2_. An innovative approach for the direct deposition of ceria (1, 3 or 5 wt.%) on NiAl-LDH, based on the precipitation of Ce^3+^ ions with 1M NaOH, was developed. The proposed method allows us to obtain the CeO_2_ phase and to preserve the NiAl layered structure by avoiding the calcination treatment. The synthesis of Au-containing samples was performed through the deposition–precipitation method. The as-prepared and WGS-tested samples were characterized by X-ray powder diffraction, N_2_-physisorption and X-ray photoelectron spectroscopy in order to clarify the effects of Au and CeO_2_ loading on the structure, phase composition, textural and electronic properties and activity of the catalysts. The reduction behavior of the studied samples was evaluated by temperature-programmed reduction. The WGS performance of Au/NiAl catalysts was significantly affected by the addition of CeO_2_. A favorable role of ceria was revealed by comparison of CO conversion degree at 220 °C reached by 3 wt.% CeO_2_-modified and ceria-free Au/NiAl samples (98.8 and 83.4%, respectively). It can be stated that tuning the properties of Au/NiAl LDH via CeO_2_ addition offers catalysts with possibilities for practical application owing to innovative synthesis and improved WGS performance.

## 1. Introduction

Among the key drivers responsible for the renewed interest in the water–gas shift (WGS) reaction is associated with the growing hydrogen production. Hydrogen is considered an efficient sustainable energy carrier and an alternative carbon-free fuel that could substitute fossil fuels in the near future. The utilization of pure hydrogen in energy conversion technologies as fuel cells is anticipated to ensure an environmentally friendly way to satisfy global energy needs [1,2]. 

The manufacture of pure hydrogen for fuel cell applications requires the consideration that the presence of CO in the produced H_2_-rich synthesis gas could irreversibly destroy the metal anode in the fuel cells. Therefore, the purification of synthesis gas by CO subtraction is of particular importance, and is commonly attained via conversion of CO by water vapor, referred to as WGS reaction (CO + H_2_O ↔ CO_2_ + H_2_, ∆H = −41.2 kJ mol^−1^). The last is conventionally applied to reduce the CO level and to enrich synthesis gas with hydrogen as well. Since WGS reaction is reversible and moderately exothermic, it is thermodynamically favored at low temperatures and kinetically at high ones; thus, the equilibrium CO conversion decreases with increasing the reaction temperature. In regard to industrial application, aiming to achieve high CO conversion, the up-to-date WGS reaction is performed through a two-stage WGS converter system using both different temperatures and catalysts. There is a high temperature shift stage (350–450 °C, Fe_2_O_3_-Cr_2_O_3_, residual CO of about 2–5%), followed by a low temperature shift stage (180–250 °C, Cu-ZnO-Al_2_O_3_, residual CO content < 1%) with interstage cooling [3,4,5,6,7].

A variety of catalysts combining noble or transition metals (Pt, Rh, Au, Pd, Fe, Cu, Ni, Co) with different oxides or mixed oxides can catalyze the WGS reaction, as described in many scientific reviews and numerous papers. The process parameters and recent advances in WGS catalysis, the design of novel and effective catalyst compositions, the preparation approaches, the nature of the support, the catalyst precursors and promoter additives (if there) as well as their impact on the performance in the WGS reaction have been debated and clarified [5,6,7,8,9]. 

Amid WGS catalysts, the supported gold ones have played a key role for more than three decades [10,11,12,13,14,15]. Because of their unique features, such as stability in oxidizing surroundings, non-pyrophority, no need of additional activation pre-treatment, appreciable WGS activity and specific electronic and structural peculiarity, these catalytic formulations have proven to be among the most auspicious options to replace the conventional WGS catalysts for small-scale applications in the low-temperature range (180–260 °C). It is also important to note that the choice of support used is of crucial importance for the WGS behavior of supported gold nanoparticles. By way of illustration, supported gold on non-reducible oxides (SiO_2_, Al_2_O_3_ or MgO) showed lower activity as opposed to those on reducible oxides (Fe_2_O_3_, CeO_2_, doped-CeO_2_, ZrO_2_, TiO_2_, etc.). These oxides significantly improve gold catalyst activity in the low-temperature WGS region [3,16,17,18]. 

Ceria (CeO_2_) is among the most extensively studied reducible supports. The considerable performance of ceria and ceria-based mixed oxides in WGS reaction is related to its high capacity to store and release oxygen by Ce^3+^/Ce^4+^ surface sites, surface and bulk oxygen vacancies and redox properties. The exceptional exchange between Ce^3+^ and Ce^4+^ oxidation states, the easy change from Ce^4+^ (CeO_2_) under oxidizing conditions to Ce^3+^ (Ce_2_O_3_) under net reducing conditions and vice versa are typical for ceria. Non-stoichiometric CeO_2−y_ can be formed by oxygen release and reduction of Ce^4+^ to Ce^3+^, with the concomitant formation of oxygen vacancies within the crystal structure. The specific features and application of CeO_2_ in catalysis have been revealed in numerous reviews and papers [19,20,21,22,23,24]. Moreover, CeO_2_ maintains high dispersion of supported metals and assists the noble metal reduction, oxidation and WGS reaction as well [19]. On the other hand, nanoscaled ceria containing high concentrations of Ce^3+^ and oxygen vacancies is considered as a preferred carrier for the Au catalysts for low-temperature WGS reaction. The first reports about the unique WGS activity of Au/CeO_2_ at low temperatures were accomplished by the Flytzani-Stephanopoulos [25] and Andreeva [26] groups. After that, many efforts have been focused on the elucidation of the effect of ceria and ceria-doped oxides on WGS performance of gold catalysts depending on the ceria particle size, surface area, surface structure, presence of dopants, etc. [10,15,27,28,29,30,31,32,33,34,35,36].

Among the various carriers of gold catalysts, layered double hydroxides (LDHs) have been stated as appropriate supports of efficient WGS catalysts [37,38,39,40]. In recent years, considering the metal availability and economic considerations, an object of research investigations has been the design of nickel-based LDHs as precursors of WGS catalysts [41,42,43,44,45,46,47]. NiAl LDHs, also called takovite-like compounds Ni_6_Al_2_(OH)_16_CO_3_·4H_2_O [48] are layered nanometric materials, which are members of a large family of natural or synthetic inorganic anionic clay-like layered compounds. They are built from a two-dimensional structure consisting of successively alternating positively charged NiAl hydroxide layers [Ni^2+^_1-*x*_Al^3+^*_x_*(OH)_2_]*^x^*^+^ and an interlayer space [A^n−^*_x_*_/*n*_·*m*H_2_O] containing charge compensating exchangeable anions (CO_3_^2−^, SO_4_^2−^, NO_3_^−^, Cl^−^, etc.) and *m* water molecules. The layered structure assumes uniform distribution of the octahedrally coordinated Ni^2+^ and Al^3+^ ions within the hydroxide sheets. One key feature of LDHs is the ability to exchange various metals in the metal hydroxide sheets, which permits tailoring of the catalyst to a specific function. Upon heating, the layered structure loses the interlayered anions and water molecules and finally forms NiAl mixed metal oxides, Ni^2+^(Al^3+^)O, characterized by nanoscaled crystal size, high surface area and well-distributed both cations in situ [49,50]. 

The incorporation of rare earth elements into the structure of the LDHs is very attractive because they can affect the catalytic, electrical and magnetic properties. However, the incorporation of cerium into NiAl hydroxide-like layers is disputed due to the large ionic radius of Ce^3+^ (1.15 Å) in comparison with Ni^2+^ (0.69 Å) and Al^3+^ (0.51 Å) [51]. This is the reason that there are few publications about the synthesis of cerium containing NiAl LDHs. Sanati and Rezvani [52] reported the synthesis of Ni/AlCe LDH by precipitation with triethylamine in an autoclave without nitrogen. The authors supposed that the Ce^3+^ ions do not fill any octahedral site in the hydroxide-like layers, beingdeposited on the LDH surface, making a separate CeO_2_ phase. Another procedure for synthesis of the Ni/AlCe LDH compound by the co-precipitation method also followed by hydrothermal treatment in the autoclave for 48 h at 120 °C was presented [53]. A successful introduction of Ce^3+^ cations into the brucite-like layers of the layered structure was testified.

An important aspect to consider is the approach of CeO_2_ synthesis. The main chemical methods for the preparation of nanoscaled ceria including homogeneous precipitation, sol-gel process, sonochemical synthesis, thermal decomposition, hydrothermal synthesis, microwave synthesis, microemulsion and solvothermal method were studied by several researchers and recently described in detail by Reni and Nesaraj [54].

Among these techniques, the precipitation method has been extensively studied because it is an unpretentious process, easy scale-up and low cost. For example, Matijević and Hsu [55] prepared sub-micron crystalline oxydicarbonate Ce_2_O-(CO_3_)_2_·H_2_O particles using cerium nitrate and urea. The sample calcined at 400 °C for 2 h converted to a cubic CeO_2_. Chen and Chen [56] synthesized CeO_2_ particles from cerium nitrate with hexamethylenetetramine (HMT), which can hydrolyze slowly to yield ammonia as an alternative to urea. The difference between both methods can be explained by the different ligands. In the Ce-HMT method, ligands are OH^−^ ions, while in the Ce-urea method, the major ligands are OH^-^ ions and CO_3_^2−^ ions. The formation of Ce_2_O-(CO_3_)_2_ rather than CeO_2_ in the Ce-urea method is the result of the competition between OH and CO_3_^2−^. It seems that the urea-based method is unsuitable for the precipitation of pure Ce^3+^-containing compounds. Zhou et al. [57] obtained CeO_2_ particles of about 4 nm from cerium nitrate and ammonia precipitant at pH value ≈ 9 under oxygen bubbling into the reactor for oxidation of the Ce^3+^ to Ce^4+^ ions. The dried at room temperature precipitates directly yielded CeO_2_ particles. The synthesis of nanocrystalline CeO_2_ powders via a carbonate precipitation method, using ammonium carbonate (AC) as the precipitant and cerium nitrate hexahydrate as a cerium source was reported [58]. It was found that the precursors synthesized at AC/Ce^3+^ molar ratio (R) of 2 < R ≤ 3 are basic carbonates (Ce(OH)CO_3_·2.5H_2_O), whereas those produced at R > 3 are ammonium cerous double carbonates ((NH_4_)*_x_*Ce(CO_3_)_1.5+*x*/2_·*y*H_2_O, *x* ≤ 1.0). The CeO_2_ powder calcined at 700 °C can be densified to ≈99% of the theoretical value using isothermal sintering at 1000 °C for 2 h. Another method is based on the preparation of CeO_2_ particles from cerium chloride and sodium hydroxide with the presence of hydrogen peroxide under various pH conditions from 6 to 12 [59]. Uekawa et al. [60] obtained 7–9 nm CeO_2_ particles starting from cerium nitrate in the polyethylene glycol solution.

The majority of the abovementioned methods are focused on the type of CeO_2_ precursors, presence of ligands and additives. In most cases, the formation of CeO_2_ is achieved after temperature treatment.

We propose a simple and innovative approach for the direct deposition of ceria on NiAl-LDH, based on the precipitation of Ce^3+^ ions with NaOH. The suggested method allows us to obtain CeO_2_ phase and to preserve the NiAl layered structure by avoiding the need for capping ligand, hydrothermal and calcination treatment. Briefly, the interaction of Ce(NO_3_)_3_·6H_2_O with alkaline solution immediately leads to the formation of Ce(OH)_3_ hydroxide precipitate, due to the extremely low solubility constant (7 × 10^−21^) [61]. In alkaline environment, Ce^3+^ ions are oxidized to hydrated Ce^4+^ ions [62], and then further hydrolyzed to form a [Ce(OH)*_x_*(H_2_O)*_y_*]^(4−x)+^ complex [63,64]. The complex is deprotonated by water molecules to CeO_2_ [62]. It is obvious that the presence of OH^−^ ions strongly affects the oxidation of Ce^3+^ to Ce^4+^ ions. With regard to this finding, NaOH was chosen for precipitation of Ce^3+^ ions on NiAl-LDH.

Regarding our recent studies on the suitability of NiAl (Ni^2+^/Al^3+^ = 2.5/1) LDH as a carrier of gold-containing WGS catalysts [44] and the unique properties of ceria, the goal of the current work was to obtain novel catalytic systems of improved WGS performance via CeO_2_ addition. A modification of NiAl with CeO_2_ was performed, aiming to affect the dispersion of gold species. The role of the CeO_2_ dopant (1, 3 or 5 wt.%) and gold presence (3 wt.%) in the studied NiAl LDHs were estimated by a comparative analysis of the catalytic and reduction behavior, phase compositions, structural and surface properties of the CeO_2_-modified and gold-containing NiAl samples, with their unmodified analogues, before and after the activity tests.

## 2. Materials and Methods

### 2.1. Reagents

All the reagents, nickel nitrate hexahydrate (Ni(NO_3_)_2_·6H_2_O), aluminum nitrate nonahydrate (Al(NO_3_)_3_·9H_2_O), cerium nitrate hexahydrate (Ce(NO_3_)_3_·6H_2_O), gold(III) chloride trihydrate (HAuCl_4_·3H_2_O), sodium carbonate anhydrous (Na_2_CO_3_) and sodium hydroxide (NaOH) were of “pro analyze” purity grade, provided by Sigma-Aldrich (Steinheim, Germany), and were used for the synthesis as received. All the solutions were prepared with fresh distilled water. 

### 2.2. Sample Preparation

#### 2.2.1. Adjusting the Procedure for NiAl-LDH Modification with CeO_2_

##### Synthesis of CeO_2_

The CeO_2_ sample was prepared by a wet chemical precipitation method using Ce(NO_3_)_3_·6H_2_O and NaOH at room temperature. An appropriate amount of nitrate salt was placed in a reaction vessel and dissolved in distilled water under vigorous stirring. The pH of nitrate solution was adjusted to a value of 12 by dropwise addition of 1M NaOH. The immediately formed suspension was treated for 2 h under constant stirring conditions (pH = 12 and room temperature). The resultant precipitate was filtered and washed with distilled water to complete the removal of NO_3_^−^ ions (testing with a solution of diphenylamine in H_2_SO_4_), then was dried at 105 °C for 20 h and designated CeO_2_-105. Subsequently, a part of the dried solid was calcined at 250 °C for 2 h and marked CeO_2_-250. The choice of calcination temperature is associated with our goal to preserve the NiAl layered structure, which was further modified with CeO_2_.

##### Synthesis of NiAl-LDH

The carbonate form of NiAl-LDH with a Ni^2+^/Al^3+^ molar ratio of 2.5/1 was obtained through the co-precipitation method at 80 °C and constant pH = 8 by the usage of two aqueous solutions: mixed 0.5M Ni-Al nitrate and a 0.9M Na_2_CO_3_ as a precipitant. 

A certain volume of distilled water was loaded into a five-necked glass reactor supplied with a stirrer, pH electrode, thermocouple and reflux condenser. The water was heated to 80 °C and pH value of 8.0 was tuned with Na_2_CO_3_ solution. The proper amounts of both mixed Ni-Al and Na_2_CO_3_ solutions were simultaneously entered into the reactor by means of two peristaltic pumps with the reactants feed flow rate of 1 L h^−1^ under stirring at 260 rpm. The gained slurry was aged for 60 min under the controlled conditions, filtered off and carefully washed with hot distilled water until the pH of the filtrate decreased to ≈6–7 and the absence of NO_3_^−^ ions. The precipitate was dried at 105 °C for 20 h, named the as-synthesized sample, and denoted as NiAl. Part of this sample was heated at 250 °C for 2 h, and coded as NiAl-250.

#### 2.2.2. Synthesis of CeO_2_-Modified NiAl-LDH

The synthesized NiAl-LDH would be further modified with CeO_2_ by precipitation of Ce(NO_3_)_3_·6H_2_O with alkaline solution. Because of that, initially, the stability of NiAl-LDH in an alkaline medium was inspected. The solid was treated with 1M NaOH solution under vigorous stirring at room temperature for 2 h. The washed sample (pH of filtrate ≈ 6–7) was dried at 105 °C and named NiAl-1MNaOH. Part of this sample was heated at 250 °C for 2 h (NiAl-1MNaOH-250).

The influence of the precipitating agent concentration was examined by precipitation of 5 wt.% CeO_2_ on NiAl-LDH using 0.1M and 1M NaOH solution. The obtained modified samples were denoted as 5CeNiAl-0.1MNaOH and 5CeNiAl-1MNaOH, respectively. After calcination at 250 °C for 2 h, they were marked as 5CeNiAl-0.1MNaOH-250 and 5CeNiAl-1MNaOH-250, respectively. 

Three CeO_2_-modified NiAl-LDH samples, containing 1, 3 and 5 wt.% CeO_2,_ were prepared by the direct deposition of ceria over the NiAl-LDH suspended in distilled water, based on the precipitation of Ce^3+^ ions with 1M NaOH, following the procedure depicted above. The synthesized samples were designated as *x*CeNiAl, where *x* represents wt.% CeO_2_, for example, 1CeNiAl.

#### 2.2.3. Deposition of Gold on the Surface of NiAl and *x*CeNiAl LDHs

Gold-comprising samples were obtained by deposition–precipitation of gold over the NiAl and *x*CeNiAl LDHs. The solids were suspended in distilled water through ultrasound. The deposition of gold (3 wt.%) was performed by simultaneous addition of aqueous solutions of 0.06 M HAuCl_4_·3H_2_O and 0.2 M Na_2_CO_3_ into the reactor at 60 °C and pH = 7.0 under stirring at 250 rpm and reactant feed flow rate of 0.15 L h^−1^. After aging under the same conditions for 60 min, the sample was filtered and carefully washed with distilled water until the absence of Cl^−^ ions. The gained Au-containing materials were dried under vacuum at 80 °C and designated as Au/NiAl and Au/*x*CeNiAl. 

Since in all studied samples, the Ni^2+^/Al^3+^ molar is the same (2.5/1) and the content of gold is also identical, for convenience, they will not be indicated in the text.

### 2.3. Sample Characterization Methods

The chemical composition of the as-synthesized materials was defined by inductively coupled plasma atomic emission spectroscopy (ICP-AES) by a JY 38 spectrometer (Horiba Jobin—Yvon, Longjumeau, France) after appropriate acid treatment. 

The phase composition of the as-synthesized and post-WGS reaction samples (spent catalysts) was determined by the powder X-ray diffraction (PXRD) technique. The PXRD patterns were obtained using a Bruker D8 Advance powder diffractometer (Bruker-AXS, Karlsruhe, Germany) employing CuK*α* radiation (U = 40 kV and I = 40 mA) and LynxEye detector (Bruker-AXS, Karlsruhe, Germany). Scans were performed for 2*θ* values from 5° to 90° with a step of 0.04° 2*θ*. The crystalline phases were identified by means of International Centre for Diffraction Data (ICDD) powder diffraction files. The unit cell parameters and mean size of the coherently scattering domains (LVol-FWHM) were obtained through the analysis of line positions and profile broadening by using the fundamental parameters peak shape description, including appropriate corrections for the instrumental broadening and diffractometer geometry with the program TOPAS V4.2 (Bruker-AXS, Karlsruhe, Germany).

The texture characteristics of the as-synthesized and spent catalysts were determined through N_2_ adsorption—desorption measurements conducted at a low temperature (−196 °C) with a Sorptomatic 1990 (Thermo Finnigan, Milan, Italy) apparatus. Prior to the measurements, the samples were degassed for 2 h at room temperature followed for 36 h at 80 °C under a vacuum. Specific surface area (*SSA*) values were calculated according to the Brunauer–Emmett–Teller (BET) method from the linear part of the N_2_ adsorption isotherms [65]. Micropore volume (*V_mic_*) values were calculated using the Dubinin–Radushkevich method [66]. The mesopore volume (*V_meso_*) and the mesopore size distribution were estimated by the Barrett, Joyner and Halenda (BJH) method [67] from the desorption branch using the Lecloux standard isotherm [68]. Total pore volume (*V_tot_*) was estimated from the N_2_ volume physisorbed for relative pressure (*p/p_0_*) of 0.99.

The temperature-programmed reduction (TPR) in the as-synthesized LDHs was accomplished in the measurement cell of a SETARAM model DSC-111 differential scanning calorimeter (SETARAM, Caluire, France). The temperature was linearly raised from 25 to 700 °C at a heating rate of 10 °C min^−1^. The TPR experiments were performed by a gas mixture of 10% H_2_ in Ar at a flow rate of 20 cm^3^ min^−1^. The selected experimental conditions are in agreement with the criteria recommended by Monti and Baiker [69] to avoid mass transfer and temperature control limitations.

The X-ray photoelectron spectra (XPS) were recorded with a VG Microtech ESCA 3000 Multilab (VG Scientific, Sussex, UK), equipped with a dual Mg/Al anode. The spectra were excited by the unmonochromatized Al K*α* source (1486.6 eV) run at 14 kV and 15 mA. The experimental procedure has been previously described [70]. The constant charging of the samples was removed by referencing all the energies to the C 1s set at 285.1 eV, arising from the adventitious carbon. Analyses of the peaks were carried out with the Casa XPS software. Atomic concentrations were calculated from peak intensity using the sensitivity factors provided by the software. The binding energy values were quoted with a precision of ±0.15 eV and the atomic percentage with a precision of ±10%.

### 2.4. WGS Activity Evaluation

The catalytic tests in WGS reaction were carried out in a flow reactor at atmospheric pressure and temperature interval 120–300 °C by stepwise increase in the reaction temperature using 0.5 cm^3^ samples in the 0.63–0.80 mm particle size range and gas feed composition of 3.37 vol.% CO, 25.01 vol.% H_2_O and 71.62 vol.% Ar with space velocity of 4000 h^−1^ (gas flow rate 2 L h^−1^). The gas flow rate was controlled by a mass flow controller AALBORG Model GFC17 (AALBORG, Orangeburg, NY, USA). The temperature controller “COMECO” Model RT1800 (COMECO, Plovdiv, Bulgaria) was used for temperature control in the reactor. The control in the thermostatic box was carried out by “COMECO” (Model RT38), aiming to prevent water vapor condensation. A syringe pump RAZEL model R-99 (Razel Scientific Instruments, a part of Mansfield Research and Development, Saint Albans, VT, USA) was used for control of the water vapor concentration. The measurements were performed within four consecutive days (about 40 h). The test of each sample includes: (i) first day—activation of the catalysts by stepwise increase in the reaction temperature in the reaction gas mixture; (ii) second day—temperature dependence of CO conversion; (iii) third day—tests at different space velocities and CO/H_2_O ratio; (iv) fourth day—repeated temperature dependence of CO conversion. The WGS activity was expressed by the degree of CO conversion after the reaching of a stationary CO conversion (at every 20 °C step) The outlet CO concentration was analyzed by a Uras 3G (Hartmann & Braun AG, Frankfurt am Main, Germany) gas analyzer. The CO conversion degree was calculated based on the inlet and outlet CO concentration.

## 3. Results and Discussions

### 3.1. Tuning the Procedure for NiAl-LDH Modification with CeO_2_

We started with the synthesis of CeO_2_ aiming to verify the literature reports that ceria can be obtained directly by precipitation of Ce^3+^ ions with alkaline solution. Part of the CeO_2_ powder prepared by the above-described synthesis procedure was additionally calcined at 250 °C, for the reason of comparison.

**Figure 1:**
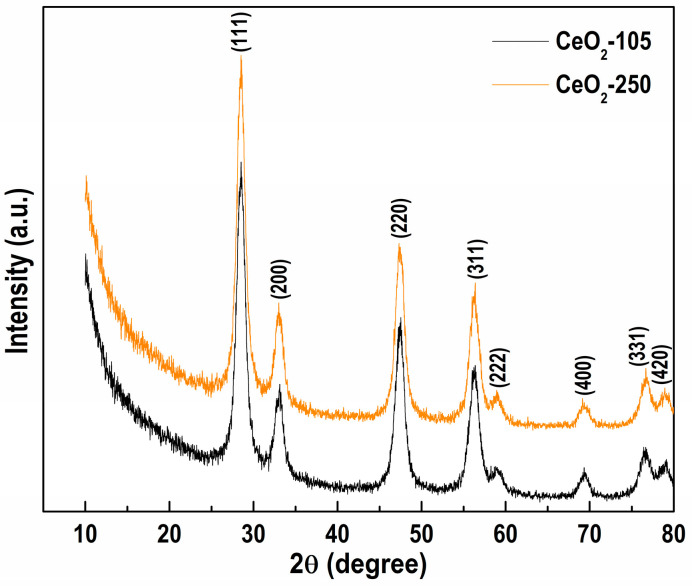
Powder X-ray diffraction (PXRD) patterns of unsupported CeO_2_, thermally treated at 105 and 250 °C.

The samples’ structural features were estimated by means of PXRD analysis (Figure 1). The patterns of dried (CeO_2_-105) and calcined CeO_2_ (CeO_2_-250) powders comprise reflections at 2*θ* = 28.5°, 33.1°, 47.5°, 56.3°, 58.9°, 69.4°, 76.6° and 78.9°, which are attributed to (111), (200), (220), (311), (222), (400), (331) and (420) planes, characteristic of the ceria phase in the cubic crystal structure of the fluorite type (ICDD-PDF file 00-034-0394). The diffractograms of both samples seem very similar in the peak angle location and intensity. The calculated unit cell parameter (*a*) and the total volume (*V*) of CeO_2_ crystal lattice (Table 1) confirm this observation, showing comparable values to those of the standard CeO_2_ oxide (ICDD-PDF file 00-034-0394). The results evidenced that we obtained a well-crystallized monophasic ceria structure in the dried as well as in the calcined samples.

Moreover, the PXRD analysis reveals comparable mean crystallite sizes (*L*) of ceria (Table 1), a little larger for the CeO_2_-250, indicating slightly improved crystallinity of the sample after the calcination at 250 °C. 

The obtained data provide experimental evidence for direct CeO_2_ synthesis by precipitation of Ce^3+^ ions using 1M NaOH without further thermal treatment.

**Figure 2:**
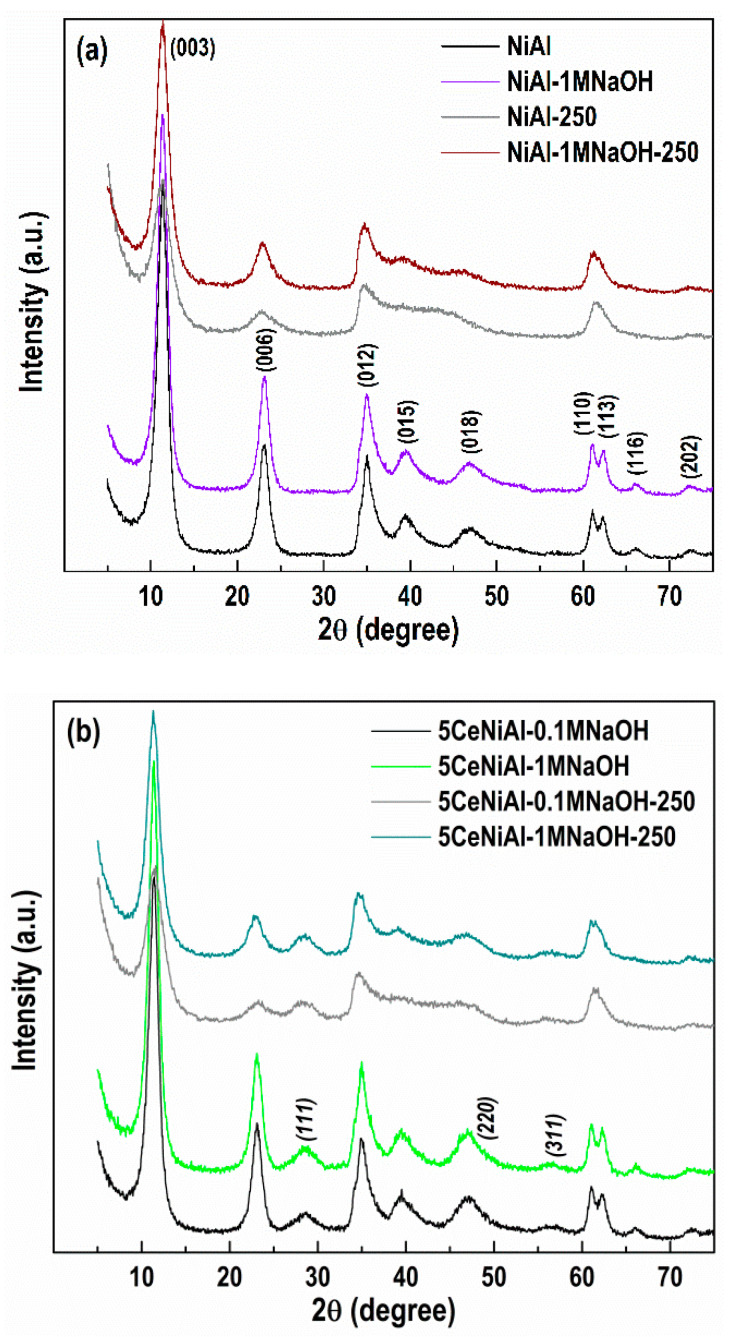
PXRD patterns of: (**a**) as-prepared NiAl, alkali treated NiAl-1MNaOH, calcined NiAl-250 and calcined alkali treated NiAl-1MNaOH-250 and (**b**) as-prepared 5CeNiAl-0.1MNaOH, and 5CeNiAl-1MNaOH and calcined 5CeNiAl-0.1MNaOH-250 and 5CeNiAl-1MNaOH-250. The diffraction lines of CeO_2_ phase are marked in italics.

The recorded PXRD spectrum of the as-synthesized NiAl sample (Figure 2a) highlights the reflections at 11.38°, 23.09°, 34.90°, 39.41°, 46.84°, 60.92°, 62.29°, 66.18° and 72.60°. These can be related to (003), (006), (012), (015), (018), (110), (113), (116) and (202) planes, corresponding to the crystal lattice of the mineral takovite (ICDD-PDF file 00-015-0087), a nickel aluminum LDH with Ni/Al = 3.0/1 molar ratio.

Considering the peak positions of the takovite structure, PXRD analysis confirms a successful synthesis of the NiAl LDH structure. Additionally, the high crystallinity of NiAl sample is pointed out by the well-resolved doublet of the reflections at 2*θ* = 60.92° and 62.29° ((110) and (113) planes) that is connected to the high degree of crystallinity [49] and good ordering of cations in the hydroxide layers [71].

The stability of NiAl-LDH in a strongly alkaline medium was examined through the treatment with 1M NaOH solution under vigorous stirring at room temperature. The PXRD patterns of NiAl-1MNaOH sample display diffraction peaks, which wholly match the positions and intensities of those of the as-prepared NiAl. PXRD characterization designates that NiAl-LDH is stable in a highly alkaline environment, since the layered structure is completely preserved and the appearance of the additional new phases is not registered as well. As can be seen, the calcination at 250 °C of NiAl and NiAl-1MNaOH (Figure 2a) induces broadening of the takovite characteristic reflections, which appear almost at the same positions as those of the as-synthesized NiAl, disclosing reduced crystalline nature of the LDH. Actually, an intermediate metastable dehydrated takovite-like (TKl) structure with reduced peak number and intensities is created. This phenomenon is attributed to the reversible removal of physisorbed water on the TKl external surface and the interlayer water, weakly bonded with charge compensating CO_3_^2−^ anions from the interlayer space [72]. Noticeably, the thermal treatment more appreciably affects the structure of the non-alkali employed sample (NiAl-250). The diffractogram of alkali operated calcined solid (NiAl-1MNaOH-250) suggests preservation of the layered structure to a greater extent than in non-alkali treated NiAl-250, confirming the stability of the NiAl sample in 1M NaOH. 

The deposition of 5 wt.% CeO_2_ on NiAl-LDH applying Ce(NO_3_)_3_·6H_2_O and two NaOH solutions (0.1M NaOH and 1M NaOH) was performed in order to optimize the conditions for NiAl modification with CeO_2_. PXRD analysis of 5CeNiAl-0.1MNaOH and 5CeNiAl-1MNaOH indicates that the usage of both NaOH solutions prompts the appearance of new diffraction lines, in addition to the TKl phase (Figure 2b). The reflections at 2*θ* = 28.3°, 47.4° and 56.2°, which are ascribed to (111), (220) and (311) lines of the cubic ceria phase, respectively, are well organized in the case of 1M NaOH application. Similarly, the PXRD study of Ce-doped NiAl samples calcined at 250 °C (5CeNiAl-0.1MNaOH-250 and 5CeNiAl-1MNaOH-250) demonstrates the co-existence of NiAl-LDH and CeO_2_ phases, also well defined when 1M NaOH is used. The comparison of PXRD patterns of dried and calcined Ce-doped NiAl samples disclosed a well-preserved layered structure when the precipitation of Ce^3+^ ions was performed with 1M NaOH. It is obvious that 1M NaOH as precipitation agent is more appropriate for ceria deposition over the NiAl-LDH because it contributes to obtaining better defined structures in comparison with the solution of lower NaOH concentration.

It may be summarized that the registration of CeO_2_ in the uncalcined samples confirms the statement that oxidation of Ce^3+^ to Ce^4+^ ions still occurs in the wet precipitate. These findings allowed us to modify the NiAl-LDH with 1, 3 and 5 wt.% CeO_2_ via Ce^3+^ ion precipitation with 1M NaOH at room temperature. Further, these samples were used as supports of deposited gold particles.

### 3.2. Chemical Analysis

The chemical composition of the obtained samples is presented in Table 2. The ICP analysis discloses that the Ni^2+^/Al^3+^ molar ratio in all the as-synthesized compositions is identical to that in the mixed NiAl nitrate solution used for the NiAl preparation. The oxide form of the components is included for a clear understanding. The gold loading in the Au-containing samples is also listed in the Table.

### 3.3. Water–Gas Shift Activity

**Figure 3:**
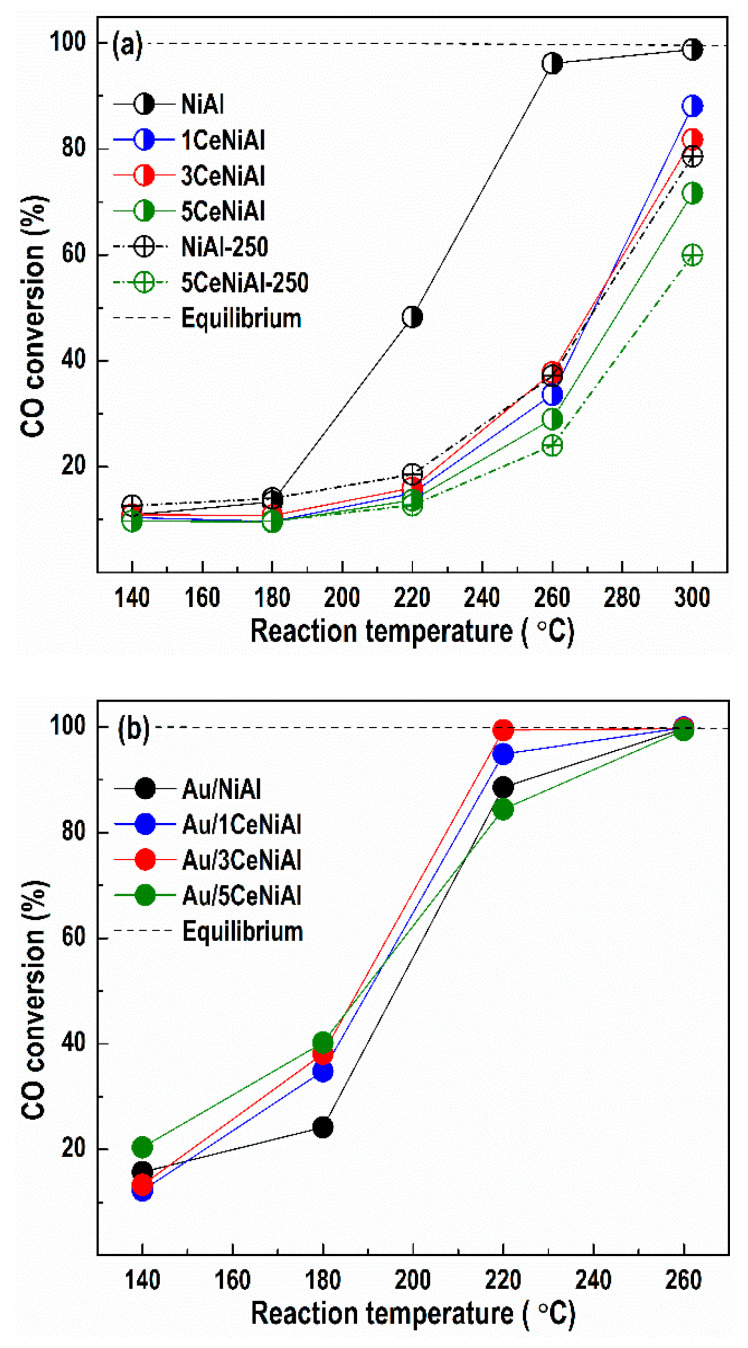
The temperature dependence of CO conversion during WGS reaction over: (**a**) NiAl and *x*CeNiAl samples and (**b**) Au/NiAl and Au/*x*CeNiAl samples. Calcined NiAl-250 and 5CeNiAl-250 are included for comparison in section (**a**).

Unlike generally accepted preliminary calcination of the catalysts, we conducted WGS activity tests directly over the as-synthesized samples aiming to preserve the layered Ni-Al structure. This concept is based on the catalytic measurements that demonstrated lower WGS activity of NiAl and 5CeNiAl after calcination even at a low temperature of 250 °C within 2 h (Figure 3a). 

When the WGS reaction starts over the uncalcined samples, the layered structure begins to decompose with the reaction temperature increase, causing formation of the NiO phase. Considering the associative reaction mechanism [44], the NiO structure represents a “precursor” for creation of the active nickel species in the redox WGS reaction. Under the reaction conditions, partial hydroxylation of the NiO surface takes place, prompting the creation of active centers on the catalyst surface, namely, Ni(OH)_2_ and NiOOH structures containing Ni^2+^ and Ni^3+^ cations, respectively. It is understandable that the creation of these species on the surface of the uncalcined sample is accomplished prior to those of the calcined NiAl sample because of structural changes that occur after calcination (see Figure 2a). This, in turn, contributes to lower catalytic activity of calcined NiAl.

In Figure 3, the catalytic activity data collected after catalyst activation are reported. As can be seen in Figure 3a, the calcined NiAl sample (NiAl-250) gains only 37.2% CO conversion at temperature of 260 °C versus the as-prepared NiAl analogue, which achieves 96.2% conversion at the same temperature. Similarly, the calcination of the CeO_2_-doped sample (5CeNiAl-250) does not contribute to the increase in WGS activity. Therefore, in order to achieve high CO conversion at lower temperatures, the usage of uncalcined samples is more favorable than calcined ones.

Another observation is that the CeO_2_ addition in the selected three concentrations does not enhance the catalytic activity of NiAl-LDH. On the other hand, the deposition of gold over the NiAl and *x*CeNiAl solids significantly improves their CO conversion, thus verifying the promoting effect of gold to activate the CO molecule. Comparison of the WGS activity of all gold-containing catalysts reveals that Au/3CeNiAl shows 99.7% CO conversion at 220 °C, i.e., CO almost reaches the equilibrium conversion degree. The rest of the catalysts demonstrate similar, but lower, activity at the same reaction temperature, following the order: Au/1CeNiAl > Au/NiAl > Au/5CeNiAl.

**Figure 4:**
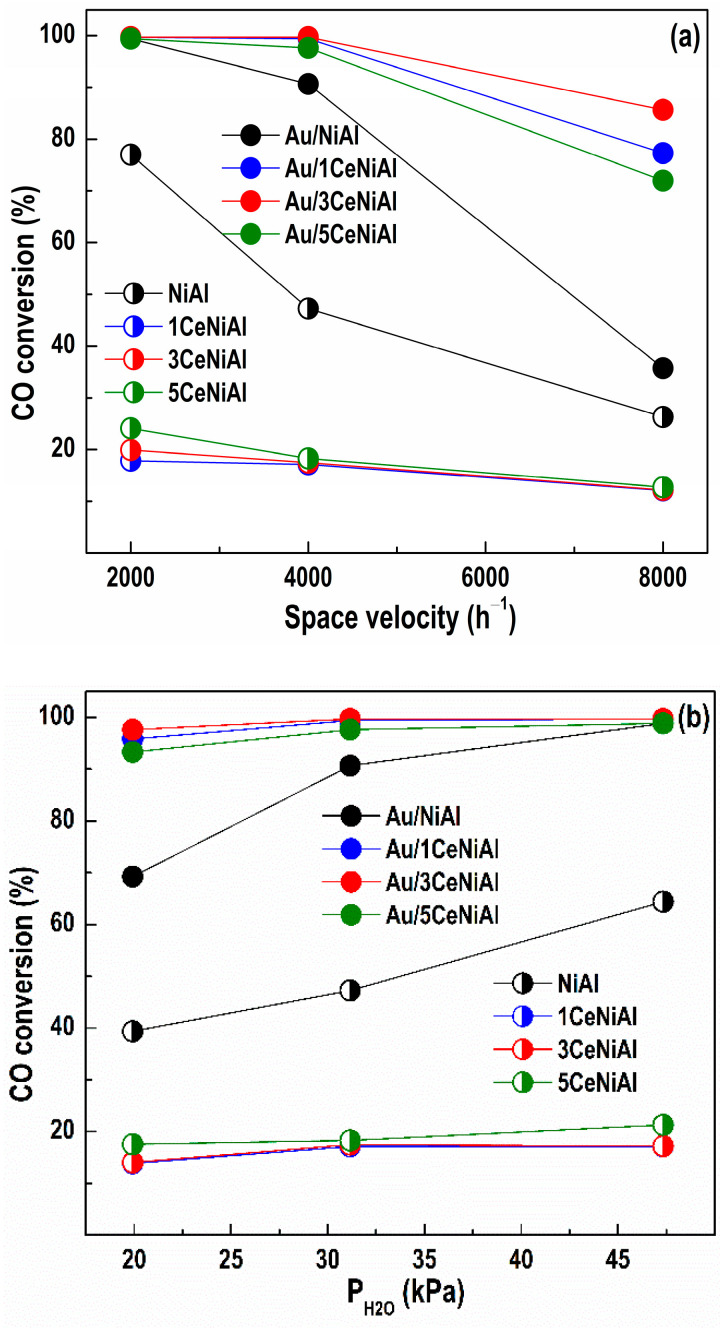
Effect of the (**a**) space velocity and (**b**) water vapor partial pressures on the degree of CO conversion at 220 °C over the studied catalysts.

The stability of the catalysts was examined under different space velocities (Figure 4a) and different water vapor partial pressures (Figure 4b). Studying the impact of the space velocities on the degree of CO conversion at 220 °C (the temperature at which maximum WGS activity is achieved over Au/3CeNiAl) in the studied catalysts represents the effect of the CO conversions as a function of the contact time. The steady-state CO conversion as a function of space velocity is demonstrated in Figure 4a. The NiAl catalyst exhibits diminution in CO conversion with the contact time decrease, while this effect is practically negligible for the Ce-modified NiAl catalysts, especially at the highest space velocity of 8000 h^−1^.

The impact of the contact time on WGS activity of Au-containing catalysts is more substantial (Figure 4a). They all display the same WGS activity at a space velocity of 2000 h^−1^. The lowering of the contact time markedly decreases the activity of the Au/NiAl catalyst, showing 36% CO conversion at 8000 h^−1^, but higher than that of the Au-free analogue (NiAl) at all studied space velocities. The increase in space velocity to 4000 h^−1^ leads to an insignificant change in CO conversion of the Au/1CeNiAl and Au/3CeNiAl catalysts and slightly decreases the CO conversion of Au/5CeNiAl. A further increase to 8000 h^−1^ induces diminution of the activity of all Au/*x*CeNiAl catalysts with 14 to 25%, namely, Au/3CeNiAl (14%) > Au/1CeNiAl (22%) > Au/5CeNiAl (25%).

The catalytic behavior in the WGS reaction also depends on the water partial pressure. The dependence of WGS activity on the water amount at 220 °C is shown in Figure 4b. The increase in water partial pressure (from 20 to 47.3 kPa) provokes an increase in CO conversion of both NiAl and Au/NiAl, more significantly in the case of the Au-containing sample (with 30%). The Ce-modified catalysts demonstrate independence from the value of the water partial pressure. Contrariwise, the CO conversion of Au-containing catalysts slightly increases as a function of the H_2_O/CO ratio. It should be stressed that the presence of the highest content of water (47.3 kPa) in the reaction mixture slightly enhances the activity of Au/*x*CeNiAl catalysts, following again in the same order: Au/3CeNiAl > Au/1CeNiAl > Au/5CeNiAl. They show good tolerance toward a high concentration of water. It is known that high H_2_O/CO ratios are typical under fuel processor conditions for low-temperature shift reactors, and the resistance to the presence of a high amount of steam could be considered as an important feature.

**Figure 5:**
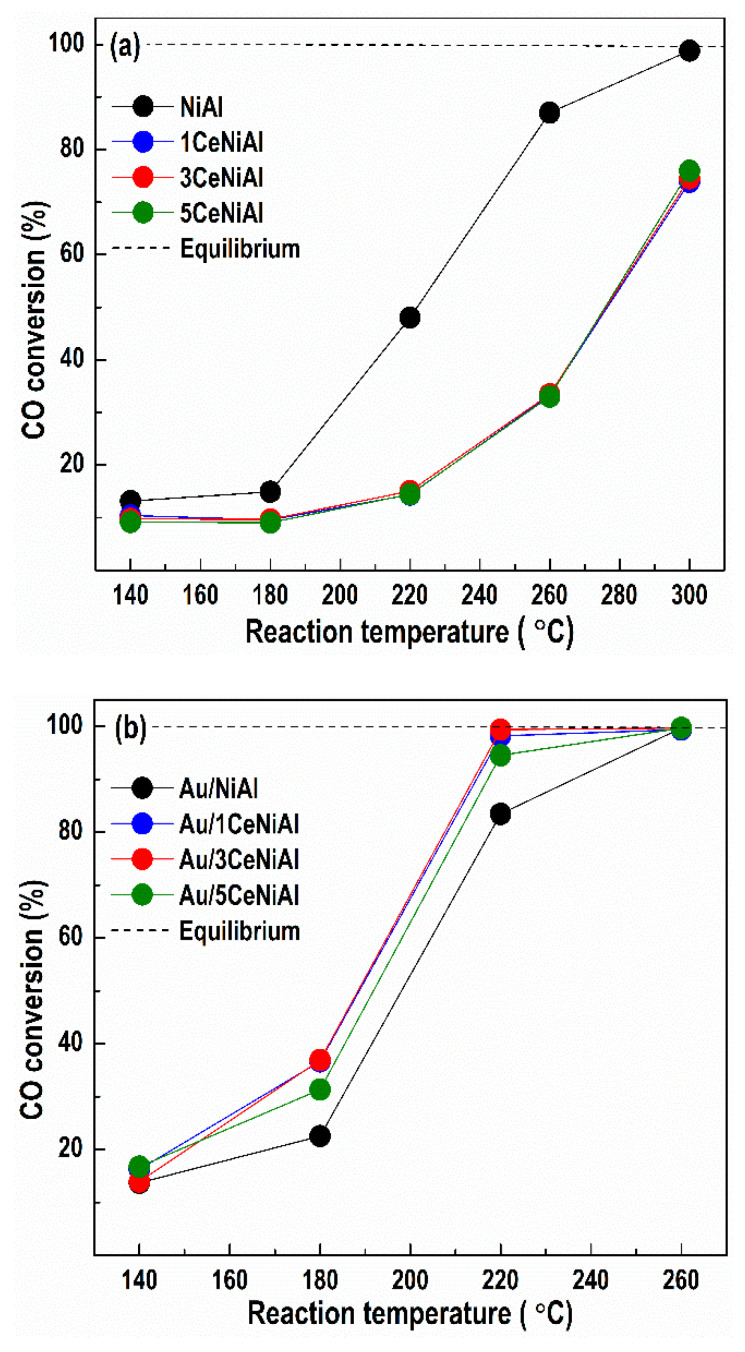
Temperature dependence of the CO conversion over the (**a**) NiAl and Ce-modified NiAl and (**b**) Au-containing NiAl and Ce-modified NiAl catalysts, studied after the stability tests.

Figure 5 illustrates the temperature dependence of the CO conversion over all catalysts studied after the stability tests (influence of space velocity and water vapor partial pressure). It should be noted that after different treatments, the NiAl catalyst demonstrates WGS activity (Figure 5a) very similar to the initial (Figure 3a), thus showing stable performance. Ce-doped NiAl catalysts also repeated the initial CO conversion (Figure 3a) regardless of the CeO_2_ content at all studied temperatures. The Au/3CeNiAl catalyst completely repeats the initial activity (Figure 5b) after the space velocity and the H_2_O/CO ratio tests. It is interesting that at 220 °C, Au/1CeNiAl and Au/5CeNiAl catalysts increase their CO conversion by 3 and 10%, respectively (Figure 5b), in comparison with the initial ones (Figure 3b). Au/NiAl exhibits the lowest activity among the gold-containing catalysts.

The results obtained disclose that gold/ceria-NiAl catalytic compositions established high CO conversion in the low-temperature range (140–260 °C) after treatment at different space velocities and H_2_O/CO ratios. Among them, the Au/3CeNiAl catalyst exhibits the highest and most stable CO conversion. As will be discussed later, this could be related to the higher gold dispersion (Table 4).

### 3.4. Structure and Phase Composition of As-Synthesized and Spent Catalysts

**Figure 6:**
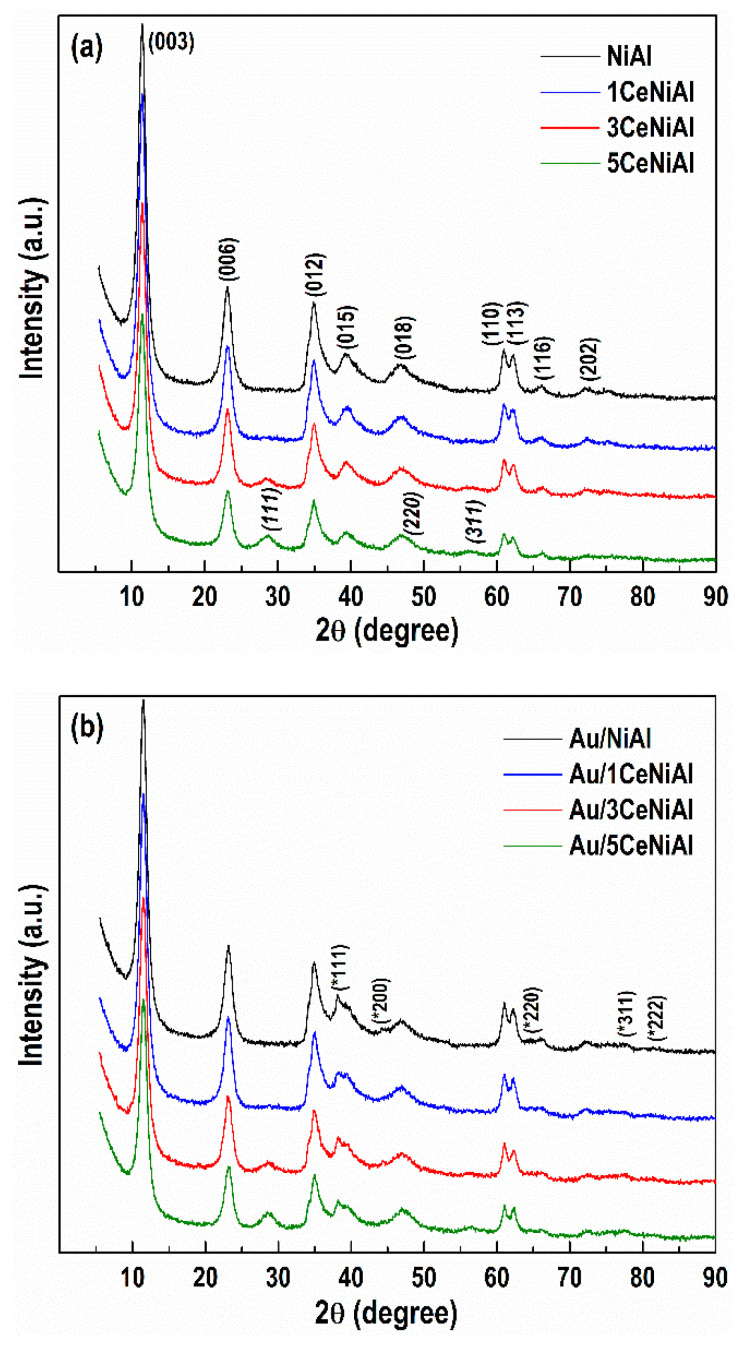
PXRD patterns of (**a**) Ce-modified NiAl samples and (**b**) Au-containing Ce-modified NiAl samples. The diffraction lines of CeO_2_ phase are marked in italics, and those of gold phase are marked by asterisks.

PXRD patterns of the as-synthesized NiAl and CeO_2_-doped NiAl samples (Figure 6a) exhibit reflections at 2*θ* = 11.38, 23.09, 34.90, 39.41, 46.84, 60.92, 62.29, 66.18 and 72.60°, which are related to (003), (006), (012), (015), (018), (110), (113), (116) and (202) planes, respectively, characteristic of the stoichiometric takovite-type structure (ICDD-PDF file 00-015-0087). 

As mentioned, additional diffraction lines at 2*θ* = 28.57, 47.44 and 56.31°, which are attributed to (111), (220) and (311) crystalline planes of cubic ceria phase (ICDD-PDF file 00-034-0394), respectively, were recorded (Figure 6a) after the precipitation of 5 wt.% CeO_2_ over the NiAl LDH (sample 5CeNiAl). The formation of the separate ceria phase over NiAl is supported by the calculated unit cell parameter *a*_CeO2_ = 5.410(6) Å and total volume *V*_CeO2_ = 158.4(5) Å^3^ of the CeO_2_ crystal lattice in 5CeNiAl (Table 3), which are identical to those of the standard CeO_2_ oxide (Table 1). The intensity and number of ceria diffraction lines decrease with the diminution of its amount to 3 wt.%, as seen in Figure 6a. The absence of a separate ceria phase in 1CeNiAl could be explained by the low CeO_2_ content of 1 wt.% in the sample. Generally, the presence of ceria lowers the crystallinity of the parent NiAl solid, which is more pronounced in the sample with the highest CeO_2_ loading, 5CeNiAl.

The deposition of gold over the NiAl and CeO_2_-doped NiAl samples (Figure 6b) is proven by the presence of the reflections at 2*θ* = 38.2, 44.4, 64.6, 77.5 and 81.7°, which are ascribed to (111), (200), (220) (311) and (222) crystalline planes, respectively, characteristic of the face centered-cubic metal phase (ICDD-PDF file 00-004-0784), in addition to the LDH phase.

PXRD patterns of NiAl and CeO_2_-doped NiAl LDHs were indexed in rhombohedral crystal symmetry with hexagonal cell setting of takovite-containing carbonate ions in the interlayer space. The analysis of the calculated unit cell parameters (*a*_TK_, *c*_TK_), total volume (*V*_TK_) and mean crystallite size (*L*_TK_) of the TKl phase discloses small differences as a function of the sample composition (Table 3). 

It is obvious that precipitation of 1 wt.% CeO_2_ over the NiAl LDH practically does not influence the parameters and crystallite size values of the sample. A very small increase in these is observed with an increase in the CeO_2_ content to 5 wt.% CeO_2_. The comparison of PXRD patterns in Figure 6b discloses that the deposition of gold over NiAl, 1CeNiAl and 3CeNiAl samples slightly decreases the crystallite size of the TKl phase in the corresponding Au-containing analogues. Additionally, all gold-supported NiAl samples possess Au^0^ particles with average sizes in the range of 14−17 nm. On the other hand, smaller Au^0^ particles of 11 nm are registered for the Au/1CeNiAl solid, outlining high dispersion of the supported gold particles (Table 2). 

The recorded specific PXRD patterns of TKl compounds in all studied solids as well as the similarity of the lattice parameters and the mean crystallite size values of the TKl phase in all the studied samples (Table 3) signify that the deposition of ceria and gold does not destroy the layered structure.

PXRD analyses performed before and after WGS tests show that under the influence of the reaction conditions, all the as-prepared samples undergo structure alterations, related to decomposition of the NiAl layered structure.

**Figure 7:**
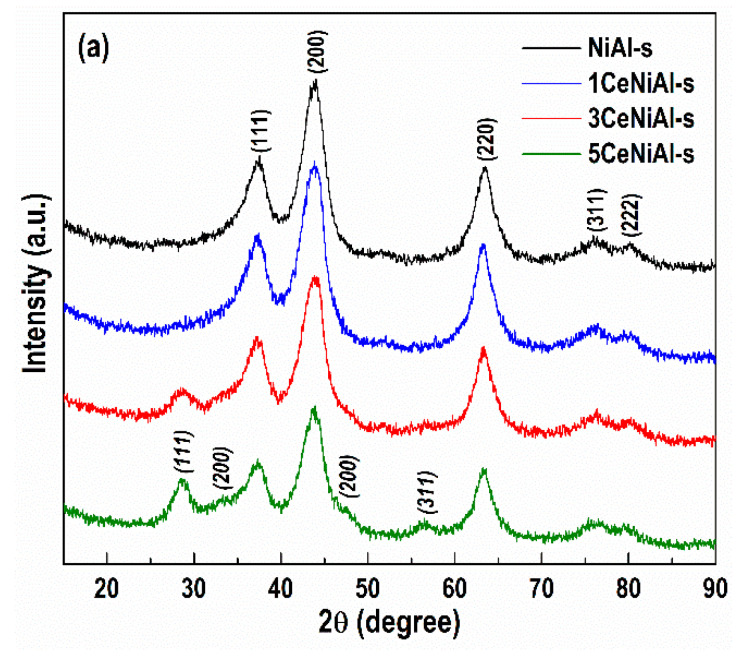

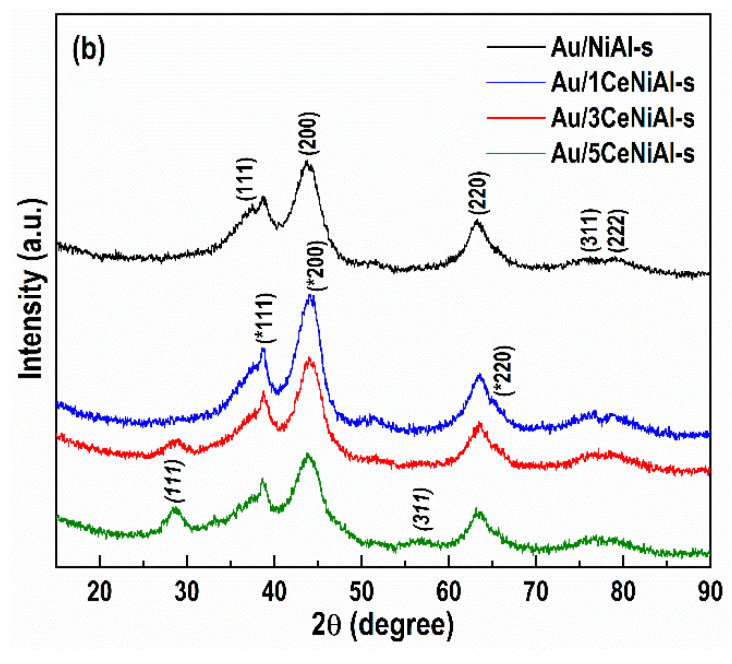
PXRD patterns of (**a**) spent Ce-doped NiAl samples and (**b**) Au-containing Ce-doped NiAl catalysts. The diffraction lines of CeO_2_ phase are marked in italics, and asterisks mark those of gold phase.

PXRD study of the spent catalysts (Figure 7) reveals reflections at 2*θ* = 37.2, 43.3, 62.9, 75.4 and 79.4, indexed as (111), (200), (220), (311) and (222) crystalline planes of the cubic NiO phase (JCPDS file 00-047-1049), respectively. The solids decompose to NiO, better organized in NiAl-s with a crystallite size of 3.09 nm (Table 4). The observed broadening of the NiO diffraction lines is ascribed to the incorporation of Al^3+^ ions into the cubic framework of NiO, leading to lattice distortion [47].

A decrease in the crystallite size of NiO with an increase in CeO_2_ from 1 to 5 wt.% was detected, outlining the role of ceria. This finding is more significant after gold deposition. The dimension of NiO phase for the gold-containing spent catalysts is in the range 2.2–2.6 nm, specifying a higher dispersion of the NiO phase in the presence of gold particles. 

The comparison of gold crystallite sizes reveals that modification of Au/NiAl catalyst with 3 wt.% CeO_2_ provides the highest dispersion of gold particles in spent Au/3CeNiAl-s catalyst (Table 4).

### 3.5. Catalyst Reduction Properties

**Figure 8:**
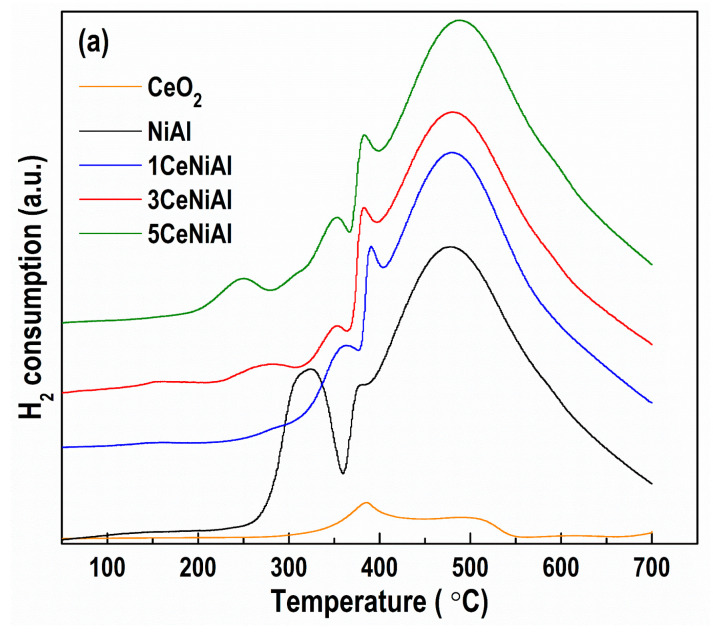

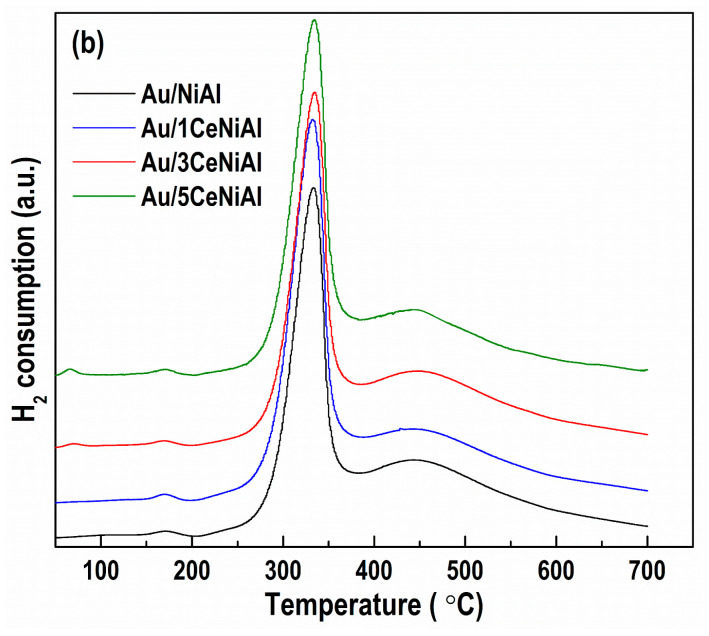
Temperature-programmed reduction (TPR) profiles of (**a**) Ce-doped NiAl samples and (**b**) Au-containing Ce-doped NiAl samples.

TPR measurements were performed to evaluate the samples’ reducibility and the effect of ceria and gold addition on oxygen mobility. Given that the experiments were carried out in as-prepared samples, the reduction process comprised thermal decomposition of takovite in the H_2_ atmosphere followed by reduction of the obtained NiO (Figure 8). In accordance with the models proposed for decomposition and reduction of NiAl LDH compounds, [73], a well-resolved peak at 320 °C and a shoulder at 385 °C from the TPR profile of NiAl sample (Figure 8a) can be attributed to the reduction of easily reducible Ni^2+^ species from a NiO phase, which contains small amounts of Al^3+^ ions. The broad peak centered at 477 °C is associated with the reduction of the Ni^2+^ species, hardly bonded to Al^3+^ ions. This represents the reduction of a quasi-amorphous non-stoichiometric spinel-type phase, which decorates the surface of the NiO particles and/or acts as their support. Addition of ceria onto NiAl caused the appearance of new low-temperature features instead of the peak at 320 °C. The intensity and position of temperature maximum (T_max_) of the peaks below 300 °C are closely related to the content of ceria. A broad peak at 250 °C was observed in the profile of the sample with 5 wt.% CeO_2_, i.e., 5CeNiAl. A weaker peak at 275 °C was registered in the pattern of 3CeNiAl, while only a shoulder around 300 °C was visible for 1CeNiAl. All these peaks could be ascribed to the reduction of easily reducible Ni^2+^ species affected by ceria in close vicinity. Observed the profile of pure ceria (lab-prepared following the same procedure as in the case of ceria-modified NiAl), a large broad peak between 350 and 550 °C with T_max_ at 380 °C can be seen and attributed to the surface ceria oxygen reduction. Numerous papers have reported experimental evidence for the role of ceria in facilitating transition metal oxide reduction, but also the effect of metal oxides to enhance surface reduction of ceria. We suggest that these low-temperature components resulted from the combined contribution of Ni species and CeO_2_ to the reducibility of the samples. Additionally, well-discernible maxima at about 350 and 380 °C were produced in the complex TPR profile with a maximum temperature in the interval from 477 (NiAl) to 487 °C (5CeNiAl). These components correspond to various types of Ni^2+^ species with different reducibility due to a complex interaction between the components of LDHs. Despite the appearance of weak low-temperature peaks, the shift to a higher temperature of reduction implies that ceria hampers reducibility, as reported recently by Swirk et al. [74]. This effect of ceria on reduction behavior could explain lower WGS activity of ceria-modified LDHs (Figure 3a).

The presence of gold remarkably boosts the redox properties of all LDHs (Figure 8b). The broad high-temperature peaks were shifted significantly towards lower temperatures. The reduction profiles were dominated by narrowed peaks with very similar intensity and identical T_max_ = 335 °C, assigned to Ni^2+^ reduction, in agreement with the well-known ability of gold to improve reducibility of metal oxides by weakening the M-O bond [25,26]. Additionally, hydrogen dissociation occurs on small metallic gold particles, and the produced active hydrogen atoms can spill over onto the support and enhance the reduction. Very weak peaks at 65 (Au/5CeNiAl) and 71 °C (Au/3CeNiAl) correspond unequivocally to ceria surface layer reduction due to the dependence of their intensity and T_max_ position on the amount of ceria. The similarity in reduction behavior, respectively in oxygen mobility, correlates well with catalytic performance.

### 3.6. N_2_ Physisorption Analysis

Knowledge of the textural characteristics of the as-prepared NiAl LDHs used as a carrier of the active species (Au) is necessary to understand the contributions of modifiers (Ce-species) and the active phase. However, this is insufficient to give an estimate of the magnitude of the impact of possible texture change on the properties and activity of the catalysts used in the WGS reaction. In addition, the influence of reaction conditions on the texture of the support (NiAl), ceria-modified support (CeNiAl) and catalyst (Au/CeNiAl) would remain hidden. Therefore, by measuring the physisorption of N_2_ at −196 °C, a textural characterization was performed not only of freshly prepared samples of NiAl, 3CeNiAl, Au/NiAl and Au/3CeNiAl but also of their pairs used in catalytic tests. From all ceria-modified NiAl supports, 3CeNiAl was chosen for characterization because the corresponding gold-containing catalyst (Au/3CeNiAl) showed the best catalytic properties.

**Figure 9:**
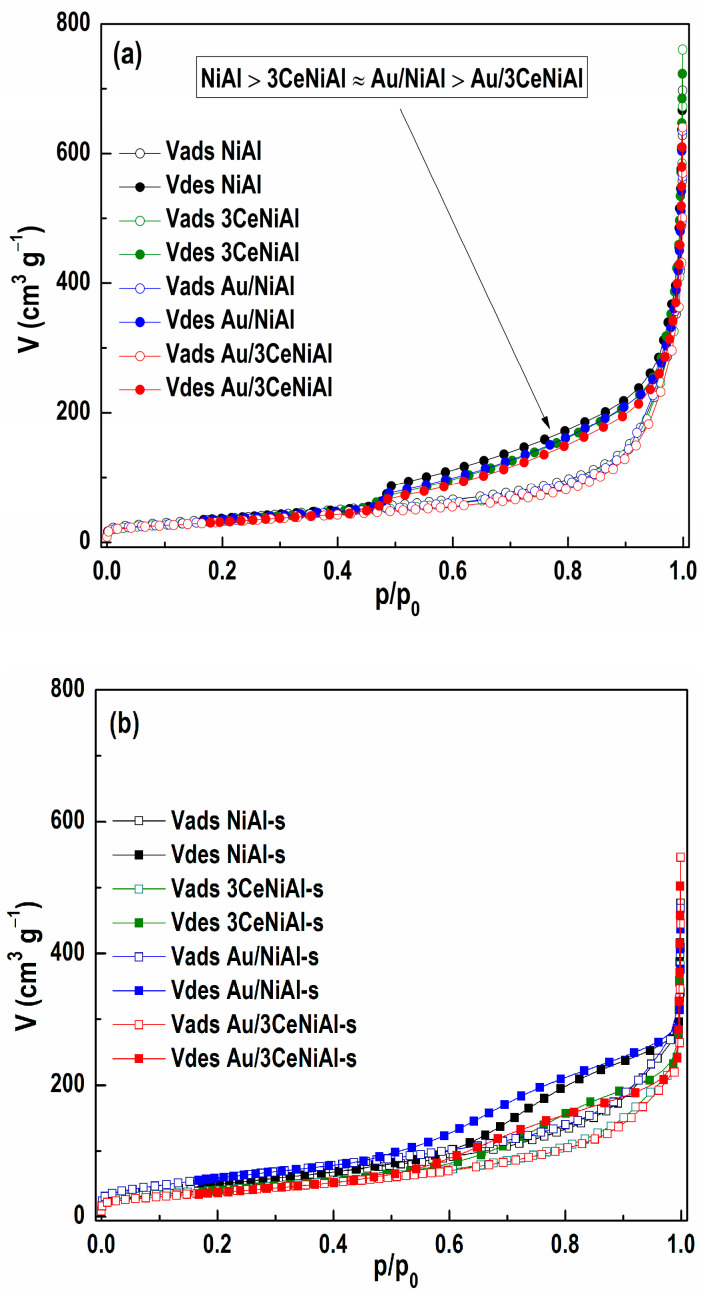
N_2_ adsorption–desorption isotherms of (**a**) as-synthesized samples and (**b**) spent catalysts.

**Figure 10:**
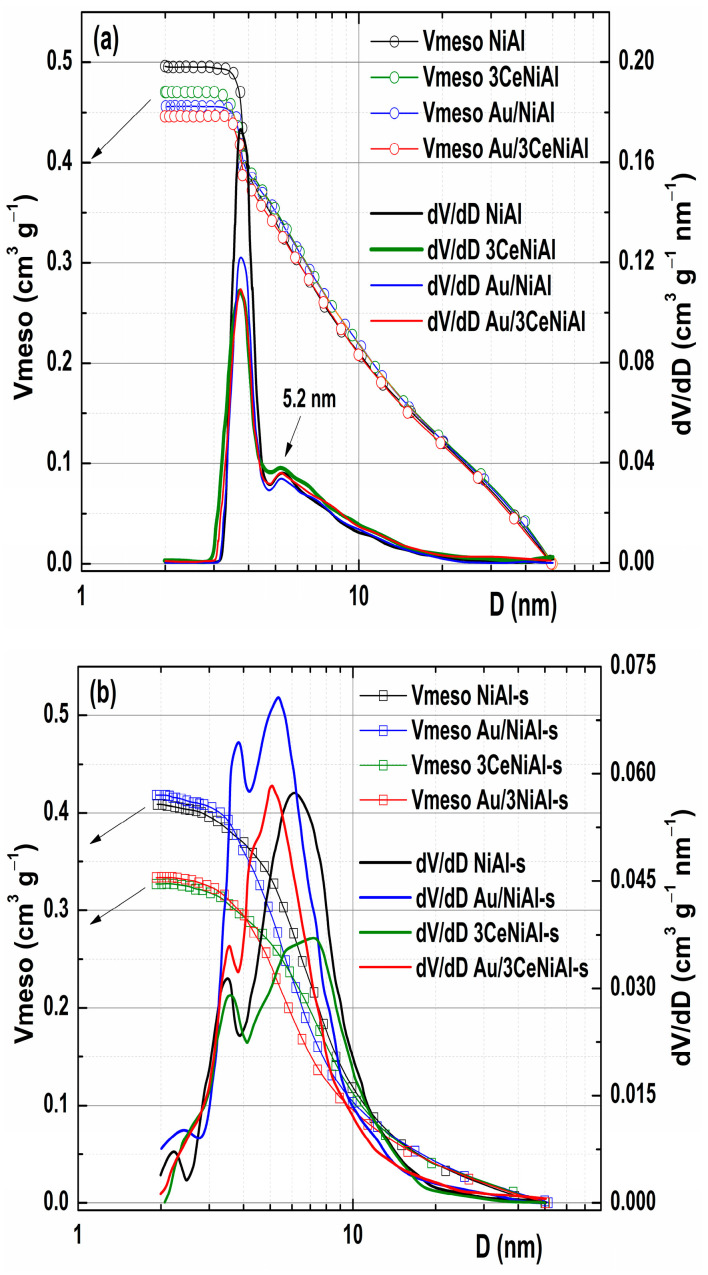
Mesopore size distribution (PSD) curves of (**a**) as-synthesized samples and (**b**) of spent catalysts.

The N_2_ isotherms of the freshly prepared and spent samples are shown in Figure 9, while the integral and differential distributions of mesopores are given in Figure 10, and all calculated textural parameters are given in Table 5.

The shape of the isotherms of all four fresh synthesized materials (Figure 9a) is remarkably similar. The adsorption part of all isotherms has the same features: (i) a limited increase in the region of low relative pressures; (ii) an increase slightly concave toward the *x*-axis with *p/p*_0_ increase, followed by an almost linear increase up to *p/p*_0_ ≈ 0.8; (iii) upraised convex towards the *p/p*_0_ axis; and (iv) a significant increase in the highest values of relative pressure. The desorption part, from the higher relative pressure side, is almost parallel to the adsorption branch, while the hysteresis loop, obviously present at *p*/*p*_0_ ≤ 0.95, and existing from highest relative pressure in all isotherms, ends at *p/p*_0_ ≈ 0.42. All the above characteristics classify the isotherms of all four materials as type II with hysteresis loop H3, according to the IUPAC nomenclature [65], or as type IIb, as proposed by Rouquerol et al. [75]. This type of isotherm with a H3 hysteresis loop is typical of many materials, e.g., clays, pigments and cements, with aggregates of plate-like particles, which possess non-rigid slit-shaped pores. 

The fact is that individually adding 3 wt.% CeO_2_ or Au causes a slight reduction in the value of all textural parameters relative to NiAl (Table 5). This change seems to be independent of the species being added. For example, desorption branches of isotherms of Au/NiAl and 3CeNiAl are literally identical, while the decrease in the specific surface area of 20 m^2^ g^−1^ for the Au/3CeNiAl catalyst compared to unmodified NiAl is almost the ideal sum of *SSA* losses with a single addition of CeO_2_ or Au (12 m^2^ g^−1^ + 9 m^2^ g^−1^). However, the similarity between the shapes of all four isotherms in Figure 9 indicates that the addition of only 3wt.% CeO_2_ or gold, and even their combinations, in a total amount of 6 wt.% does not substantially alter the pore system present in unmodified NiAl LDH. For the mesoporous region, shown in Figure 10, this is undoubtedly true for the 50–4 nm segment. The overlap of all four curves of the mesopore size distribution (PSD) is almost complete. Positions of maxima on these curves, at 3.8 and 5.2 nm, are also identical.

The difference in peak intensity is usually a consequence of the different pore volume of that diameter. However, the peak of the smaller diameter on the PSD curve does not have to originate from the pores that actually exist, but from the so-called “tensile strength effect” [76], which corresponds to the hysteresis curve closing position at about 0.42 *p/p*_0_. Thus, the difference in the peak intensities at 3.8 nm may also be due to the existence of different pore contents in the segment below that diameter value.

Exposure of freshly prepared samples to reaction conditions leads to recognizable alteration in the shape of the isotherms of all four materials (Figure 9b), as well as to a significant change in their texture parameters (Table 5), but not all in the same way. First, although the deviation of the desorption branch from the almost parallel tracking of the adsorption branch is evident in all isotherms of the used samples, this deviation is unevenly expressed. A kind of a hump on the desorption branch in the region of relative pressure 0.9 to 0.6 is evident and most noticeable for spent Au/NiAl-s and NiAl-s samples, and barely visible for ceria modified support (3CeNiAl-s). Further, for all spent samples compared to freshly prepared ones, two of the textural parameters, *SSA* and *V_mic_*, increase, while the other two, *V_meso_* and *V_tot_*, decrease. At the same time, the intensities of change are not nearly the same for samples that have the same tendency. For example, an increase in *SSA* reaches over 70% for the Au/ and only 17% for the 3CeNiAl-s support. Additionally, a similar trend of non-equal increase in micropore volume can be observed.

Comparing only the results of the final mesoporous volume of all four materials, it is obvious that they are grouped in two pairs: the first made of Au/NiAl and its support NiAl (0.418 vs. 0.409 cm^3^ g^−1^), and the second made of Au/3CeNiAl and its ceria-modified support (0.333 vs. 0.327 cm^3^ g^−1^). This type of grouping is also recognized when BET surface area values are taken into account. Although the addition of gold to NiAl and exposure to WGS reaction conditions increased the *SSA* value by 30 m^2^ g^−1^ compared to NiAl-s, while its addition to 3CeNiAl caused almost no changes in the *SSA* compared to 3CeNiAl-s (reduction by 4 m^2^ g^−1^), the differences between these pairs are obvious.

The size distribution of the mesopores of materials used in the WGS reaction (Figure 10b) shows the existence of two peaks for all materials, just as for freshly prepared samples, although with a significantly changed ratio of peak intensity, namely, the peak corresponding to the smaller diameter decreased significantly and had a smaller contribution to the overall PSD profile in all samples. It is interesting to note that the positions of the maximum values corresponding to the pores of larger diameter for the used unmodified and cerium-modified support were moved to higher values by 0.8 and 2 nm, respectively.

The applied reaction conditions, mainly the temperatures, but also gaseous reactants and products lead to the decomposition of materials based on NiAl LDH, contributing to the change of their textural characteristics, regardless of whether they are supports or catalysts. The values of all textural parameters (*SSA*, *V_mic_* and *V_meso_*) are smaller in Au/3CeNiAl compared to unmodified Au/NiAl, regardless of whether pairs of freshly prepared samples or a sample used in catalytic tests are analyzed.

Therefore, based on the determined values of texture characteristics, it can be said with certainty that the improved activity of Au/3CeNiAl compared to Au/NiAl cannot be caused by a change in texture properties due to modification of NiAl support by ceria.

### 3.7. X-ray Photoelectron Spectroscopy (XPS)

The oxidation-reduction characteristic of the WGS reaction requires information to be obtained for the components’ oxidation states on the catalysts surface of the most active Au/3CeNiAl catalysts compared to NiAl, 3CeNiAl and Au/NiAl samples in their as-prepared and postreaction state (spent state). The correlation between the surface species state and WGS activity was searched for. Attention was also given to another activity-determining factor, such as relative dispersion of Au and Ce over NiAl-LDH, and the Ni/Al ratio before and after WGS reaction.

The binding energy (BE) values of the as-prepared samples are summarized in Table 6. The main Ni2p_3/2_ peak of the bare NiAl is characterized by the binding energy (BE) of 855.4 eV and the shake-up peak, 6 eV apart from the main Ni 2p_3/2_ peak, corresponding to the Ni^2+^ state. Bearing in mind the nature of NiAl LDH, namely, the layered NiAl hydroxide sheet structure, the BE value is attributed to Ni(OH)_2_ (855.3–856.6 eV) [77,78,79,80].

**Figure 11:**
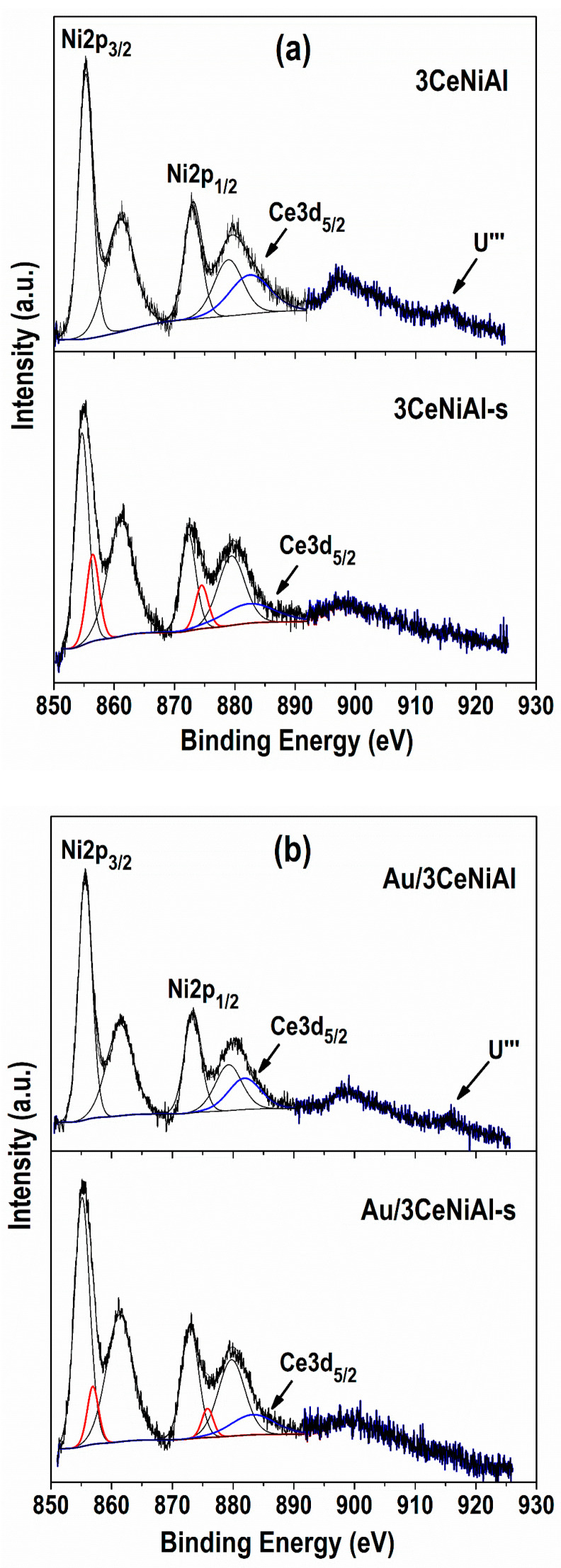
Ni 2p- and Ce 3d-photoelectron regions of the studied samples: (**a**) 3CeNiAl as-prepared and spent; (**b**) Au/3CeNiAl as-prepared and spent. The Ni^3+^ oxidation state is colored in red. The blue contour line outlines the Ce 3d signal.

The modification of NiAl material with 3 wt.% CeO_2_ does not cause changes in the layered structure of 3CeNiAl, as is documented by XRD data. In contrast, the gold deposition in both Au/NiAl and Au/3CeNiAl shifts Ni 2p_3/2_ peaks toward higher BEs by 0.3 eV, namely, 855.7 eV (Table 6 and Figure 11) more than the ±0.15 eV experimental accuracy. A similar shift in the gold-containing samples is observed in the Al 2p binding energy (Table 6), assigned to Al^3+^ species. The shifts of Ni 2p and Al 2p binding energies reveal a stronger bond in the layered hydroxide structure. This statement is in agreement with the small lowering of the TKl phase mean crystallite size (*L*_TK_) disclosed by XRD (Table 3). Through the curve fitting of the Ni 2p region in the spent catalysts, a lowering of BE for Ni^2+^ oxidation state compared to as-synthesized samples (Figure 11 and Table 7) is detected. Moreover, a second component at a higher binding energy of 856.7 ± 0.3 eV in all catalysts is identified, which is attributed to Ni^3+^, likely as Ni−OOH species [44,78]. The lower Ni^2+^ BEs reveal changes in the NiAl structure due to WGS reaction, namely, destroying of the NiAl layered hydroxide structure and interaction between Al and Ni in mixed NiAlO oxide structures [81]. This was also suggested by PXRD analysis of spent catalysts. The smaller shift (0.2 eV) observed in the Ni2p_3/2_ of the bare NiAl catalyst as compared to the larger shifts observed for the CeNiAl, the AuNiAl and the AuCeNiAl (0.5–0.6 eV) implies the significant impact of CeO_2_ and gold promotion on Ni^2+^ state after the reaction is run. 

In addition, a reciprocal effect of gold and ceria on the corresponding binding energy values is observed. Indeed, the main Au 4f_7/2_ peak at 84.5 eV of the as-prepared Au/3CeNiAl sample discloses a slight shift as compared to 84.7 eV of the Au/NiAl. Additionally, the main Ce 3d_5/2_ peak of the Au/3CeNiAl is detected at 881.8 eV, whereas it is detected at 882.1 eV in 3CeNiAl. It is known that the binding energy at 84.5 eV ± 0.5 eV of Au 4f_7/2_ is typical of metallic gold, and the Ce 3d_5/2_ BE value corresponds to the Ce^4+^ oxidation state [36]. Obviously, the simultaneous Au and Ce presence makes their interactions with NiAl weaker, thus suggesting their greater reactivity in the Au/3CeNiAl catalyst during redox WGS reaction. The BEs of the Au 4f_7/2_ peak in the spent Au/NiAl and Au/3CeNiAl catalysts are the same. The variation of the Au 4f_7/2_ binding energy of the spent catalysts with respect to the as-synthesized samples (Table 6 and Table 7) is within the experimental error, confirming that the gold oxidation state is preserved after WGS reaction. The surface concentration of gold on all of the as-prepared samples is the same (0.4 at.%), and it diminishes in spent catalysts (0.2 at.%).

The careful inspection of the Ce 3d photoelectron regions indicates (Figure 11) that in both 3CeNiAl and Au/3CeNiAl samples in their as-prepared state, the detected highest binding energy component is situated at 915.3 and 915.7 eV, respectively. This component is signed as U‴, typical of the Ce^4+^ oxidation state. The poor quality of the Ce 3d spectra due to the overlap with the Ni 2p and to the low CeO_2_ content (3 wt.%) did not allow a reliable fitting. However, the position of the main Ce 3d_5/2_ component and a rough estimate of the Ce at.% = 0.5 from the entire Ce 3d region partially overlapping with the Ni 2p_3/2_ were obtained. It is well known that the intensity of U‴ satellite gives information about Ce^3+^ contribution, by relating it to the total Ce 3d area [82,83]. Unfortunately, in the present spectra, variations in the intensity of the U‴ peak are hardly detectable; therefore, no conclusive statement about the partial reduction of Ce^4+^ to Ce^3+^ induced by gold can be made [28]. However, concerning the as-prepared samples, the Ce 3d_5/2_ BE shifting of 0.4 eV observed in Au/3CeNiAl as compared to the Au-free sample could be indicative of an Au-Ce^3+^ bond formed by the strain effect as claimed by a theoretical study based on density functional theory (DFT) calculations for an Au/CeO_2_ system [84]. The data in Table 7 show that the Ce 3d_5/2_ position is the same in both CeO_2_-modified NiAl-LDHs after catalytic tests (3CeNiAl-s and Au/3CeNiAl-s). The visible change is the disappearance of the U‴ peaks, confirming that CeO_2_ undergoes reduction to Ce^3+^ ions during the WGS reaction.

The associative reaction mechanism of WGS over Au/NiAl catalysts was proven in our previous papers, involving redox Ni^2+^ ↔ Ni^3+^ transition on the catalyst surface as well as adsorption and activation of the CO molecule on Au particles [44,47]. The availability of nickel in both Ni^2+^ and Ni^3+^ oxidation states on the catalyst surface contributes to the high activity of the Au/NiAl catalyst in the studied temperature range. The role of reversible redox Ni^2+^ ↔ Ni^3+^ transition implies evaluation of the Ni^3+^/Ni^2+^ ratio, which is presented in Table 7. The values clearly disclose that ceria addition increases the Ni^3+^/Ni^2+^ ratio 1.72 times, while gold presence decreases it. Evidently, the gold keeps the catalyst surface more reduced, which is in correlation with the higher activities of Au/NiAl and Au/3CeNiAl compared to NiAl. The oxidized surface of the 3CeNiAl samples can be related to the lowest WGS activity of this catalyst.

**Figure 12:**
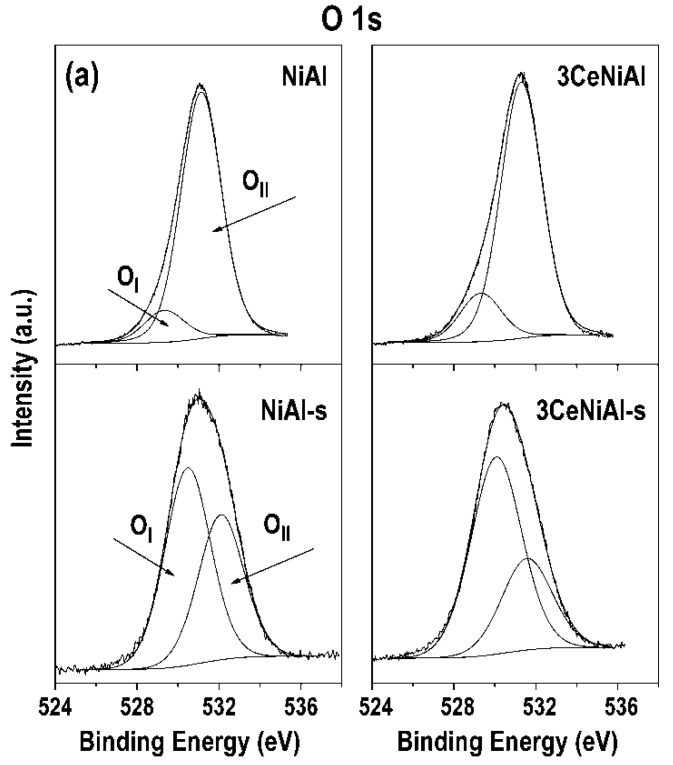

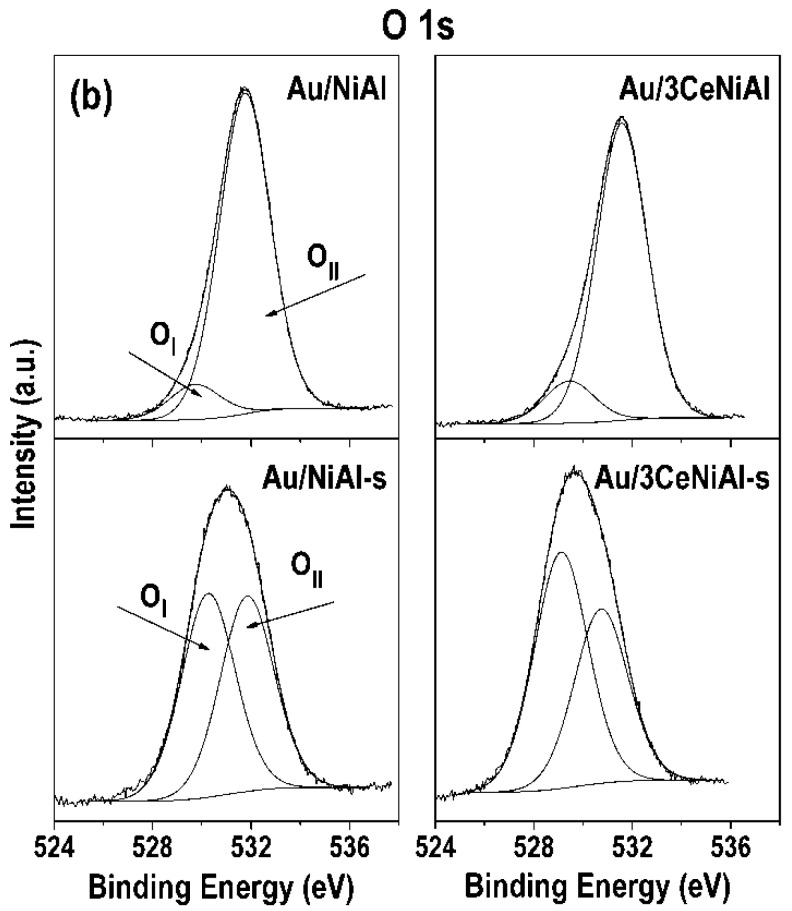
O 1s-photoelectron regions of the as-synthesized and spent samples: (**a**) NiAl and 3CeNiAl; (**b**) Au/NiAl and Au/3CeNiAl.

The review of the O 1s photoelectron regions after the curve fitting (Figure 12) shows that the spectra of the as-prepared catalysts consist of two components, namely, less intense low energy peaks centered between 529.3 and 529.7 eV and more intense higher energy peaks between 531.1 and 531.7 eV (Table 7). The low energy peaks are attributed to lattice oxygen named as O_I_ associated with the NiAl layered structure [81,85] and oxygen in the CeO_2_ lattice [86,87,88] in 3CeNiAl and Au/3CeNiAl samples. The high energy peaks recognized as surface adsorbed oxygen are named O_II_. The intensity of these peaks is significant compared to O_I_ peaks, because they belong to chemisorbed oxygen in CO_3_^2−^/OH groups from the NiAl hydroxide layer and intercalated water molecules from interlayer space [81,89] and hydroxyl groups of pure CeO_2_ [86,87,88,90]. In addition, the shifting of the O_II_ component toward higher BEs in the Au-containing samples (Au/NiAl and Au/3CeNiAl) suggests a strong interaction of metallic gold with oxygen, producing a decrease in the electronic charge of these oxygen species. A similar effect was observed in relation to the Ni 2p and Al 2p shifting mentioned above (Table 6). These data confirm the formation of a stronger bond in the layered hydroxide structure under the influence of gold. Moreover, the O_II_ contribution predominates over O_I_ and could be explained by the preserved NiAl layered structure shown by XRD analysis. The O 1s spectrum undergoes significant changes in the spent catalysts due to the redox conditions and reaction temperature. The intensity of all O_II_ peaks decreases, and O_I_ peaks become more intense (Figure 12). The BEs of lattice oxygen peaks (O_I_) are moved 0.6–1.2 eV to higher BEs (Table 7). In this case, the lattice oxygen originates from the NiAlO structure, unreduced CeO_2_ and partially reduced cerium in CeO_2-x_, namely, 529.8–530 eV [86,87], oxygen in Ni–OOH [44,91] and last but not the least, oxygen vacancies (O_x_^−^) in the matrix of metal oxides, which are usually registered in the range of 529.9−531.1 eV [92,93,94].

Genty et al. stated [89] that the mobility of surface oxygen species plays an important role in the catalytic activity in oxidation reactions. The oxygen vacancies are important for the adsorption of oxygen species [85] from H_2_O vapor in the case of WGS reaction. In this connection, the number of oxygen vacancies in the spent catalysts are calculated by the integrated area ratios of O_II_/(O_II_ + O_I_) (Table 7) by analogy to Lu et al. [85]. The data show that Au addition increases the oxygen vacancies, implying the presence of more active vacant oxygen on the catalyst surface of the Au/NiAl catalyst compared to NiAl as well as in the Au/3CeNiAl catalyst compared to 3CeNiAl. This fact correlates with higher activities of the Au-containing catalysts. The role of oxygen vacancies in facilitating the dissociation of water, considered the most difficult step in the WGS reaction, is highlighted in the literature [95,96,97]. It would also be relevant to point out that 3CeNiAl has the smallest number of oxygen vacancies, and its WGS activity is worse than that of unmodified NiAl. The relative dispersion of Au and Ce over NiAl-LDH and the Ni/Al ratio before and after WGS reaction was evaluated from XPS-derived atomic concentration (Table 8).

The gold amount is obviously the same in both as-synthesized and spent samples; however, the atomic ratio values decrease by more than half on the surface of the spent catalysts. The low values cannot be connected with poorer dispersion due to Au^0^ particle agglomeration; this is due to the XRD data (Table 3 and Table 4) displaying that after WGS reaction, the average gold particle size decreases considerably by 1.65 times in Au/NiAl-s and 2.93 times in Au/3CeNiAl-s. So, the reason for this observation can be attributed to the subsurface sinking of the gold, which is not completely visible for the XPS technique.

The ceria relative dispersion also diminishes in the spent CeO_2_-modified catalysts. The diminution is stronger in 3CeNiAl-s; in fact, the Au presence makes this negligible, and only 8.8% in the most active Au/3CeNiAl-s. The estimation of the Ni/Al ratio indicates that the bulk chemical composition in as-synthesized samples, Ni/Al = 2.5, is not the same on the surface. The reaction conditions provoke Ni enrichment of the surface in all spent catalysts. This is explained by the reconstruction of the NiAl layered structure, leading to its destruction and interaction between Ni and Al forming a mixed NiAlO structure.

## 4. Conclusions

Studying the role of the CeO_2_ dopant in the phase compositions, structural, textural and surface properties, reduction behavior and WGS activity of gold-containing NiAl LDHs allows the following conclusions to be made:

(i) The developed innovative approach for modification by ceria allows us to preserve the NiAl layered structure and to obtain a CeO_2_ phase with a good crystallinity in a relatively short time by a one-pot method, thus avoiding the calcination treatment, which simplifies the catalyst preparation procedure.
(ii) The modification of NiAl LDHs with CeO_2_ neither improves reducibility nor enhances the WGS efficiency; however, the simultaneous presence of gold and ceria has a beneficial effect.
(iii) It can be deduced that hydrogen production via WGS reaction is affected by the amount of ceria in the Au/NiAl catalyst.
(iv) The addition of 3 wt.% CeO_2_ to the Au/NiAl catalyst provides the highest dispersion of gold particles in the spent catalyst (Au/3CeNiAl-s) and contributes to a good WGS performance—highest activity and significant stability.

## Figures and Tables

**Table 1:** Structural characteristics of the synthesized CeO_2_ samples.

Sample	*a* (Å)	*V* (Å^3^)	*L* (nm)
CeO_2_-105	5.4211(10)	159.32(9)	7.27(6)
CeO_2_-250	5.4185(9)	159.09(8)	7.55(6)
CeO_2_-standard	5.4113	158.46	-

**Table 2:** Sample notation and chemical composition of the as-synthesized samples.

Sample	Chemical Composition (wt.%)				Ni^2+^/Al^3+^ Molar Ratio
	NiO	CeO_2_	Al_2_O_3_	Au	
NiAl	78.55	-	21.45	-	2.5
1CeNiAl	77.76	1.0	21.24	-	2.5
3CeNiAl	76.19	3.0	20.81	-	2.5
5CeNiAl	74.62	5.0	20.38	-	2.5
Au/NiAl	76.30	-	20.70	3.0	2.5
Au/1CeNiAl	75.43	0.97	20.60	3.0	2.5
Au/3CeNiAl	73.90	2.91	20.19	3.0	2.5
Au/5CeNiAl	72.38	4.85	19.77	3.0	2.5

**Table 3:** Structural characteristics of the as-synthesized samples.

Sample	*a*_TK_ (Å)	*c*_TK_ (Å)	*V*_TK_ (Å^3^)	*L*_TK_ (nm)	*L*_CeO2_ (nm)	*L*_Au_ (nm)
NiAl	3.0333(6)	22.964(13)	182.98(13)	9.90	-	-
1CeNiAl	3.0334(6)	22.958(13)	182.98(13)	9.80	-	-
3CeNiAl	3.0336(6)	22.974(12)	183.07(12)	10.10	-	-
5CeNiAl	3.0338(6)	22.972(13)	183.11(13)	10.20	2.16	-
Au/NiAl	3.0343(7)	22.994(12)	183.34(12)	8.05	-	14.0
Au/1CeNiAl	3.0355(6)	23.026(11)	183.75(12)	9.10	1.90	11.0
Au/3CeNiAl	3.0339(6)	23.020(12)	183.49(13)	9.30	1.82	17.0
Au/5CeNiAl	3.0353(6)	23.017(13)	183.65(13)	10.10	2.18	16.0
* Takovite	3.0250	22.5950	179.06	-	-	-

* ICDD 00-015-0087.

**Table 4:** Mean crystallite size of NiO, CeO_2_ and Au in the spent samples.

Sample	*L*_NiO_ (nm)	*L*_CeO2_ (nm)	*L*_Au_ (nm)
NiAl-s	3.09	-	-
1CeNiAl-s	3.08	n.d.*	-
3CeNiAl-s	2.98	3.10	-
5CeNiAl-s	2.78	3.50	-
Au/NiAl-s	2.65	-	8.5
Au/1CeNiAl-s	2.63	n.d.	11.8
Au/3CeNiAl-s	2.41	4.95	5.8
Au/5CeNiAl-s	2.24	3.65	9.2

* n.d.—not detected

**Table 5:** Textural parameters.

Sample	*SSA* (m^2^ g^−1^)	*V_mic_* (m^3^ g^−1^)	*V_meso_* (m^3^ g^−1^)	*V_tot_* (m^3^ g^−1^)
NiAl	138	0.048	0.496	0.575
NiAl-s	191	0.058	0.409	0.417
3CeNiAl	126	0.042	0.474	0.569
3CeNiAl-s	147	0.053	0.327	0.367
Au/NiAl	129	0.043	0.456	0.596
Au/NiAl-s	221	0.072	0.418	0.466
Au/3CeNiAl	118	0.041	0.446	0.534
Au/3CeNiAl-s	143	0.047	0.333	0.350

**Table 6:** Binding energies (eV) of the main elements’ peaks and the oxygen species contribution in as-synthesized samples.

Sample	Ni 2p_3/2_	Al 2p	O 1s Position (eV) and Contribution (%) *		Au 4f_7/2_	Ce 3d_5/2_
			O_I_	O_II_		
NiAl	855.4	74.2	529.3(11)	531.1(89)		
Au/NiAl	855.7	74.5	529.7(10)	531.7(90)	84.7	
3CeNiAl	855.3	74.2	529.3(16)	531.3(84)		882.2
Au/3CeNiAl	855.7	74.5	529.4(12)	531.6(88)	84.5	881.8

* The values in parentheses refer to the atomic percentage of the oxygen species.

**Table 7:** X-ray photoelectron spectra (XPS) analysis data of the spent catalysts.

Catalyst	Ni 2p_3/2_ Position (eV) and Contribution (%)		Ni^3+^/Ni^2+^ Ratio	O 1s Position (eV) and Contribution (%)		O_II_/(O_II_+O_I_)	Au 4f_7/2_	Ce3d_5/2_
	Ni^2+^	Ni^3+^		O_I_	O_II_			
NiAl-s	855.2(90)	856.8(10)	0.11	530.5(58)	532.1(42)	0.42		
Au/NiAl-s	855.2(93)	856.9(7)	0.08	530.3(51)	531.8(49)	0.49	84.4	
3CeNiAl-s	854.7(84)	856.4(16)	0.19	530.1(68)	531.6(32)	0.32		881.9
Au/3CeNiAl-s	855.1(92)	856.9(8)	0.09	529.1(58)	531.7(42)	0.42	84.4	881.9

**Table 8:** Relative dispersion of Au and Ce and Ni/Al ratio before and after WGS reaction.

Catalyst	Au/(Ni + Al)	Au/(Ce + Ni + Al)	Ce/(Ni + Al)	Ni/Al
NiAl				0.49
NiAl-s				0.51
Au/NiAl	0.0088			0.47
Au/NiAl-s	0.0040			0.54
3CeNiAl			0.0140	0.47
3CeNiAl-s			0.0077	0.49
Au/3CeNiAl		0.0089	0.0113	0.45
Au/3CeNiAl-s		0.0041	0.0103	0.50

## Data Availability

The data presented in this study are available in the article.

## References

[1] Baykara S.Z. (2018). Hydrogen: A brief overview on its sources, production and environmental impact. Int. J. Hydrog. Energy.

[2] Sazali N. (2020). Emerging technologies by hydrogen: A review. Int. J. Hydrog. Energy.

[3] Ratnasamy C., Wagner J.P. (2009). Water gas shift catalysis. Catal. Rev. Sci. Eng..

[4] LeValley T.L., Richard A.R., Fan M. (2014). The progress in water gas shift and steam reforming hydrogen production technologies—A review. Int. J. Hydrog. Energy.

[5] Reddy G.K., Smirniotis P.G. (2015). Water Gas Shift Reaction: Research Developments and Applications.

[6] Chen W.-H., Chen C.-Y. (2020). Water gas shift reaction for hydrogen production and carbon dioxide capture: A review. Appl. Energy.

[7] Ebrahimi P., Kumar A., Khraisheh M. (2020). A review of recent advances in water-gas shift catalysis for hydrogen production. Emergent Mater..

[8] Pal D.B., Chand R., Upadhyay S.N., Mishra P.K. (2018). Performance of water gas shift reaction catalysts: A review. Renew. Sust. Energy Rev..

[9] Cavalcanti F., Schmal M., Giudici R., Alves R. (2019). A catalyst selection method for hydrogen production through Water-Gas Shift Reaction using artificial neural networks. J. Environ. Manag..

[10] Burch R. (2006). Gold catalysts for pure hydrogen production in the water–gas shift reaction: Activity, structure and reaction mechanism. Phys. Chem. Chem. Phys..

[11] Tao F., Ma Z. (2013). Water–gas shift on gold catalysts: Catalyst systems and fundamental studies. Phys. Chem. Chem. Phys..

[12] Reina T.R., González M.C., Palma S., Ivanova S., Odriozola J.A., Ma Z., Dai S. (2014). Twenty years of golden future in the water-gas shift reaction. Heterogeneous Gold Catalysts and Catalysis.

[13] Odabasi C., Günay M.E., Yildirim R. (2014). Knowledge extraction for water gas shift reaction over noble metal catalysts from publications in the literature between 2002 and 2012. Int. J. Hydrog. Energy.

[14] Carter J.H., Hutchings G.J. (2018). Recent advances in the gold-catalyzed low-temperature water-gas shift reaction. Catalysts.

[15] Tabakova T. (2019). Recent advances in design of gold-based catalysts for H_2_ clean-up reactions. Review article. Front. Chem..

[16] Sandoval A., Gomez-Cortes A., Zanella R., Diaz G., Saniger J.M. (2007). Gold nanoparticles: Support effects for the WGS reaction. J. Mol. Catal. Chem..

[17] Liu X.Y., Wang A., Zhang T., Mou C.-Y. (2013). Catalysis by gold: New insights into the support effect. Review. Nano Today.

[18] Yang M., Flytzani-Stephanopoulos M. (2017). Design of single-atom metal catalysts on various supports for the low temperature water-gas shift reaction. Catal. Today.

[19] Trovarelli A. (1996). Catalytic Properties of Ceria and CeO_2_-Containing Materials. Catal. Rev. Sci. Eng..

[20] Trovarelli A., Trovarelli A. (2002). Structural properties and nonstoichiometric behavior of CeO_2_. Catalysis by Ceria and Related Materials.

[21] Polychronopoulou K., Kalamaras C.M., Efstathiou A.M. (2011). Ceria-Based Materials for Hydrogen Production Via Hydrocarbon Steam Reforming and Water-Gas Shift Reactions. Recent Pat. Mater. Sci..

[22] Sun C., Li H., Chen L. (2012). Nanostructured ceria-based materials: Synthesis, properties, and applications. Energy Environ. Sci..

[23] Montini T., Melchionna M., Monai M., Fornasiero P. (2016). Fundamentals and catalytic applications of CeO_2_‑based materials. Chem. Rev..

[24] Schmitt R., Nenning A., Kraynis O., Korobko R., Frenkel A.I., Lubomirsky I., Haile S.M., Rupp J.L.M. (2020). A review of defect structure and chemistry in ceria and its solid solutions. Chem. Soc. Rev..

[25] Fu Q., Weber A., Flytzani-Stephanopoulos M. (2001). Nanostructured Au-CeO_2_ catalysts for low-temperature water-gas shift. Catal. Lett..

[26] Andreeva D., Idakiev V., Tabakova T., Ilieva L., Falaras P., Bourlinos A., Travlos A. (2002). Low-temperature water–gas shift reaction over Au/CeO_2_ catalysts. Catal. Today.

[27] Luengnaruemitchai A., Osuwan S., Gulari E. (2003). Comparative studies of low temperature water–gas shift reaction over Pt/CeO_2_, Au/CeO_2_, and Au/Fe_2_O_3_ catalysts. Catal. Commun..

[28] Tabakova T., Boccuzzi F., Manzoli M., Sobczak J.W., Idakiev V., Andreeva D. (2006). A comparative study of nanosized IB/ceria catalysts for low-temperature water-gas shift reaction. Appl. Catal. A Gen..

[29] Karpenko A., Leppelt R., Plzak V., Cai J., Chuvilin A., Schumacher B., Kaiser U., Behm R. (2007). Influence of the catalyst surface area on the activity and stability of Au/CeO_2_ catalysts for the low- temperature water gas shift reaction. Top. Catal..

[30] Abdel-Mageed A.M., Kucěrova G., Bansmann J., Behm R.J. (2017). Active Au species during the low-temperature water gas shift reaction on Au/CeO_2_: A time-resolved operando XAS and DRIFTS study. ACS Catal..

[31] Lenite B.A., Galletti C., Specchia S. (2011). Studies on Au catalysts for water gas shift reaction. Int. J. Hydrog. Energy.

[32] Andreeva D., Tabakova T., Ilieva L., Trovarelli A., Fornasiero P. (2013). Ceria-based gold catalysts: Synthesis, properties and catalytic performance for the WGS and PROX processes. Catalysis by Ceria and Related Materials.

[33] Yi N., Flytzani-Stephanopoulos M., Wu Z., Overbury S.H. (2015). Gold/ceria: The making of a robust catalyst for fuel processing and hydrogen production. Catalysis by Materials with Well-Defined Structures.

[34] Reina T.R., Ivanova S., Centeno M.A., Odriozola J.A. (2016). The role of Au, Cu & CeO_2_ and their interactions for an enhanced WGS performance. Appl. Catal. B Environ..

[35] Fu X.-P., Guo L.-W., Wang W.-W., Ma C., Jia C.-J., Wu K., Si R., Sun L.-D., Yan C.-H. (2019). Direct identification of active surface species for the water–gas shift reaction on a gold–ceria catalyst. J. Am. Chem. Soc..

[36] Tabakova T., Ivanov I., Zanella R., Karakirova Y., Sobczak J.W., Lisowski W., Kaszkur Z., Ilieva L. (2020). Unraveling the effect of alumina-supported Y-doped ceria composition and method of preparation on the WGS activity of gold catalysts. Int. J. Hydrog. Energy.

[37] Santos J.L., Reina T.R., Ivanova S., Centeno M.A., Odriozola J.A. (2017). Gold promoted Cu/ZnO/Al_2_O_3_ catalysts prepared from hydrotalcite precursors: Advanced materials for the WGS reaction. Appl. Catal. B Environ..

[38] Santos J.L., Reina T.R., Ivanov I., Penkova A., Ivanova S., Tabakova T., Centeno M.A., Idakiev V., Odriozola J.A. (2018). Multicomponent Au/Cu-ZnO-Al_2_O_3_ catalysts: Robust materials for clean hydrogen production. Appl. Catal. A Gen..

[39] Meng Y., Chen Y., Zhou X., Pan G., Xia S. (2020). Experimental and theoretical investigations into the activity and mechanism of the water-gas shift reaction catalyzed by Au nanoparticles supported on Zn-Al/Cr/Fe layered double hydroxides. Int. J. Hydrog. Energy.

[40] Xia S., Fang L., Meng Y., Zhang X., Zhang L., Yang C., Ni Z. (2020). Water-gas shift reaction catalyzed by layered double hydroxides supported Au-Ni/Cu/Pt bimetallic alloys. Appl. Catal. B Environ..

[41] Fuentes E.M., Faro A.C., Silva T.F., Assaf J.M., Rangel M.C. (2011). A comparison between copper and nickel-based catalysts obtained from hydrotalcite-like precursors for WGSR. Catal. Today.

[42] Gabrovska M., Idakiev V., Tenchev K., Nikolova D., Edreva-Kardjieva R., Crisan D. (2013). Catalytic performance of Ni-Al layered double hydroxides in CO purification processes. Russ. J. Phys. Chem. A.

[43] Fuentes E.M., Aires F.J.C.S., Prakash S., Faro A.C., Silva T.F., Assaf J.M., Rangel M.C. (2014). The effect of metal content on nickel-based catalysts obtained from hydrotalcites for WGSR in one step. Int. J. Hydrog. Energy.

[44] Gabrovska M., Tabakova T., Ivanov I., Kovacheva D. (2019). Water-gas shift reaction over gold deposited on NiAl layered double hydroxides. React. Kinet. Mech. Cat..

[45] Meza-Fuentes E., Rodriguez-Ruiz J., Solano-Polo C., Rangel M.C., Faro A. (2020). Monitoring the structural and textural changes of Ni-Zn-Al hydrotalcites under heating. Thermochim. Acta.

[46] Xia S., Dai T., Meng Y., Zhou X., Pan G., Zhang X., Ni Z. (2020). Low temperature water-gas shift reaction catalyzed by hybrid NiO@NiCr elayered double hydroxides: Catalytic property, kinetics and mechanism investigation. Phys. Chem. Chem. Phys..

[47] Gabrovska M., Ivanov I., Nikolova D., Kovacheva D., Tabakova T. (2021). Hydrogen production via water-gas shift reaction over gold supported on Ni-based layered double hydroxides. Int. J. Hydrog. Energy.

[48] Bish D., Brindley G. (1977). A reinvestigation of takovite, a nickelaluminium hydroxycarbonate of the pyroaurite group. Am. Mineral.

[49] Cavani F., Trifirò F., Vaccari A. (1991). Hydrotalcite-type anionic clays: Preparation, properties and applications. Catal. Today.

[50] Clause O., Goncalves Coelho M., Gazzano M., Matteuzzi D., Trifirò F., Vaccari A. (1993). Synthesis and thermal reactivity of nickel-containing anionic clays. Appl. Clay Sci..

[51] Shannon R. (1976). Revised effective ionic radii and systematic studies of interatomic distances in halides and chaleogenides. Acta Crystallogr. A.

[52] Sanati S., Rezvani Z. (2018). Co-intercalation of acid Red-27/sodium dodecyl sulfate in a Ce-containing Ni–Al-layered double hydroxide matrix and characterization of its luminescent properties. J. Mol. Liq..

[53] Golovin S.N., Yapryntsev M.N., Ryltsova I.G., Veligzhanin A.A., Lebedeva O.E. (2020). Novel cerium‑containing layered double hydroxide. Chem. Pap..

[54] Reni M.L., Nesaraj A.S. (2020). Preparative methods and recent technological applications of ceria -based nanostructured catalyst materials in chemical and other fields-a review. Mater. Res. Innov..

[55] Matijević E., Hsu W.P. (1987). Preparation and properties of monodispersed colloidal particles of lanthanide compounds. I. Gadolinium, Europium, Terbium, Samarium, and Cerium (lll). J. Colloid Interf. Sci..

[56] Chen P.L., Chen I.W. (1993). Reactive cerium (IV) oxide powders by the homogeneous precipitation method. J. Am. Ceram. Soc..

[57] Zhou X.D., Huebner W., Anderson H.U. (2002). Room-temperature homogeneous nucleation synthesis and thermal stability of nanometer single crystal CeO_2_. Appl. Phys. Lett..

[58] Li J.-G., Ikegami T., Wang Y., Mori T. (2002). Reactive Ceria Nanopowders via Carbonate Precipitation. J. Am. Ceram. Soc..

[59] Yamashita M., Kameyama K., Yabe S., Yoshida S., Fujishiro Y., Kawai T., Sato T. (2002). Synthesis and microstructure of ceria doped ceria as UV filters. J. Mater. Sci..

[60] Uekawa N., Ueta M., Wu Y.J., Kakegawa K. (2002). Synthesis of CeO_2_ spherical fine particles by homogeneous precipitation method with polyethylene glycol. Chem. Lett..

[61] Zhou X.D., Huebner W., Anderson H.U. (2003). Processing of nanometer-CeO_2_ particles. Chem. Mater..

[62] Djuričić B., Pickering S. (1999). Nanostructured cerium oxide: Preparation and properties of weakly agglomerated powders. J. Eur. Ceram. Soc..

[63] Hirano M., Kato E. (1999). Hydrothermal synthesis of nanocrystalline cerium (IV) oxide powders. J. Am. Ceram. Soc..

[64] Chen H.-I., Chang H.-Y. (2005). Synthesis of nanocrystalline cerium oxide particles by the precipitation method. Ceram. Int..

[65] Thommes M., Kaneko K., Niemark A.V., Olivier J.P., Rodriguez-Reinoso F., Rouquerol J., Sing K.S.W. (2015). Physisorption of gases, with special reference to the evaluation of surface area and pore size distribution (IUPAC Technical Report). Pure Appl. Chem..

[66] Dubinin M.M. (1975). Physical Adsorption of Gases and Vapors in Micropores. Prog. Surf. Membr. Sci..

[67] Barrett E.P., Joyner L.G., Halenda P.P. (1951). The determination of pore volume and area distributions in porous substances. I. computations from nitrogen isotherms. J. Am. Chem. Soc..

[68] Lecloux A., Pirard J.P. (1979). The importance of standard isotherms in the analysis of adsorption isotherms for determining the porous texture of solids. J. Colloid Interface Sci..

[69] Monti D.A.M., Baiker A. (1983). Temperature-programmed reduction. Parametric sensitivity and estimation of kinetic parameters. J. Catal..

[70] Russo M., La Parola V., Testa M.L., Pantaleo G., Venezia A.M., Gupta R.K., Bordoloi A., Bordoloi B. (2020). Structural insight in TiO_2_ supported CoFe catalysts for Fisher Tropsch synthesis at ambient pressure. Appl. Catal. A Gen..

[71] Benito P., Labajos F., Rives V. (2006). Microwave-treated layered double hydroxides containing Ni^2^^+^ and Al^3^^+^: The effect of added Zn^2^^+^. J. Solid State Chem..

[72] Tichit D., Medina F., Coq B., Dutartre R. (1997). Activation under oxidizing and reducing atmospheres of Ni-containing layered double hydroxides. Appl. Catal. A Gen..

[73] Trifirò F., Vaccari A., Clause O. (1994). Nature and properties of nickel-containing mixed oxides obtained from hydrotalcite-type anionic clays. Catal. Today.

[74] Świrk K., Rønning M., Motak M., Beaunier P., Da Costa P., Grzybek T. (2019). Ce-and Y-Modified Double-Layered Hydroxides as Catalysts for Dry Reforming of Methane: On the Effect of Yttrium Promotion. Catalysts.

[75] Rouquerol F., Rouquerol J., Sing K.S.W., Llewellyn P., Maurin G. (2014). Adsorption by Powders and Porous Solids Principles, Methodology and Applications.

[76] Groen J.C., Peffer L.A.A., Pérez-Ramırez J. (2003). Pore size determination in modified micro- and mesoporous materials. Pitfalls and limitations in gas adsorption data analysis. Micropor. Mesopor. Mat..

[77] Kim K.S., Winograd N. (1974). X-ray photoelectron spectroscopic studies of nickel-oxygen surfaces using oxygen and argon ion-bombardment. Surf. Sci..

[78] Löchel B.P., Strehblow H.H. (1984). Breakdown of passivity of nickel by fluoride: II. Surface analytical studies. J. Electrochem. Soc..

[79] Grosvenor A.P., Biesinger M.C., Smart R.S.C., McIntyre N.S. (2006). New interpretations of XPS spectra of nickel metal and oxides. Surf. Sci..

[80] Hu X., Li P., Zhang X., Yu B., Lv C., Zeng N., Luo J., Zhang Z., Song J., Liu Y. (2019). Ni-Based Catalyst Derived from NiAl layered double hydroxide for vapor phase catalytic exchange between hydrogen and water. Nanomaterials.

[81] Zhao S., Yi H., Tang X., Gao F., Yu Q., Zhou Y., Wang J., Huang Y., Yang Z. (2016). Enhancement effects of ultrasound assisted in the synthesis of NiAl hydrotalcite for carbonyl sulfide removal. Ultrason. Sonochem..

[82] Shyu J.Z., Otto K., Watkins W.L.H., Graham G.W. (1988). Characterization of Pd/γ-alumina catalysts containing ceria. J. Catal..

[83] Wang L., Meng F. (2013). Oxygen vacancy and Ce^3+^ ion dependent magnetism of monocrystal CeO_2_ nanopoles synthesized by a facile hydrothermal method. Mater. Res. Bull..

[84] Pan Y., Nilius N., Freund H.-J., Paier J., Penschke C., Sauer J. (2013). Titration of Ce^3+^ Ions in the CeO_2_(111) Surface by Au Adatoms. Phys. Rev. Lett..

[85] Lu B., Zhuang J., Du J., Gu F., Xu G., Zhong Z., Liu Q., Su F. (2019). Highly dispersed Ni nanocatalysts derived from NiMnAl-hydrotalcites as high-performing catalyst for low-temperature syngas methanation. Catalysts.

[86] Praline G., Koel B.E., Hance R.L., Lee H.-I., White J.M. (1980). X-Ray photoelectron study of the reaction of oxygen with cerium. J. Electron Spectros. Relat. Phenom..

[87] Laachir A., Perrichon V., Badri A., Lamotte J., Catherine E., Lavalley J.C., El Fallah J., Hilaire L., Le Normand F., Quéméré E. (1991). Reduction of CeO_2_ by hydrogen. Magnetic susceptibility and Fourier-transform infrared, ultraviolet and X-ray photoelectron spectroscopy measurements. J. Chem. Soc. Faraday Trans..

[88] Pereira A., Blouin M., Pillonnet A., Guay D. (2014). Structure and valence properties of ceria films synthesized by laser ablation under reducing atmosphere. Mater. Res. Express.

[89] Genty E., Brunet J., Poupin C., Casale S., Capelle S., Massiani P., Siffert S., Cousin R. (2015). Co-Al mixed oxides prepared via LDH route using microwaves or ultrasound: Application for catalytic toluene total oxidation. Catalysts.

[90] Ingo G.M., Paparazzo E., Bagnarelli O., Zacchetti N. (1990). XPS studies on cerium, zirconium and yttrium valence states in plasma-sprayed coatings. Surf. Interface Anal..

[91] Mansour A.N., Melendres C.A. (1994). Characterization of Electrochemically Prepared γ-NiOOH by XPS. Surf. Sci. Spectra.

[92] Wang X., Wang X., Di Q., Zhao H., Liang B., Yang J. (2017). Mutual effects of fluorine dopant and oxygen vacancies on structural and luminescence characteristics of F doped SnO_2_ nanoparticles. Materials.

[93] Tu Y., Chen S., Li X., Gorbaciova J., Gillin W.P., Krause S., Briscoe J. (2018). Control of oxygen vacancies in ZnO nanorods by annealing and their influence on ZnO/PEDOT: PSS diode behavior. J. Mater. Chem. C.

[94] Jain S., Shah J., Negi N.S., Sharma C., Kotnala R.K. (2020). Nickel substituted oxygen deficient nanoporous lithium ferrite based green energy device hydroelectric cell. J. Alloys Compd..

[95] Rodriguez J.A., Wang X., Liu P., Wen W., Hanson J.C., Hrbek J., Perez M., Evans J. (2007). Gold nanoparticles on ceria: Importance of O vacancies in the activation of gold. Top. Catal..

[96] Vindigni F., Manzoli M., Damin A., Tabakova T., Zecchina A. (2011). Surface and inner defects in Au/CeO_2_ WGS catalysts: Relation between Raman properties, reactivity and morphology. Chem. Eur. J..

[97] Laguna O.H., Dominguez M.I., Romero-Sarria F., Odriozola J.A., Centeno M.A., Ma Z., Dai S. (2014). Role of oxygen vacancies in gold oxidation catalysis. Heterogeneous Gold Catalysts and Catalysis.

